# A large orca from the Lower Pleistocene of the Southern North Sea Basin pushes back the origin of macropredatory behavior in the oceanic dolphins (Odontoceti, Delphinidae)

**DOI:** 10.7717/peerj.21890

**Published:** 2026-11-10

**Authors:** Hidde Bakker, Klaas Post, Dirk K. Munsterman, Jeroen P.A. Hoekendijk, Giovanni Bianucci

**Affiliations:** 1 Natural History Musuem Rotterdam, Rotterdam, Zuid-Holland, Netherlands; 2 Netherlands Organisation for Applied Scientific Research—Geological Survey of the Netherlands, Utrecht, Netherlands; 3 Arcadis Nederland B.V., Arnhem, Gelderland, Netherlands; 4 Dipartimento di Scienze della Terra, Università di Pisa, Pisa, Italy

**Keywords:** Paleontology, Gelasian, Cetacea, Toothed whales, Killer whales

## Abstract

The evolutionary history of the biggest extant top predator of our planet, the killer whale *Orcinus orca*, remains poorly understood. Currently, the fossil record of the genus *Orcinus* includes only a single formally recognized extinct species, the small-bodied (3.5 meters) piscivorous *Orcinus citoniensis* from the Pliocene of Cetona, Italy. Here we describe and refer to *Orcinus* sp., five delphinid fossil remains from the Pleistocene of the southern North Sea, consisting of two fragmentary crania, an incomplete mandible and two atlas-axis cervical complexes. Regression analyses based on extant *Orcinus orca* measurements and 3D model comparisons indicate an estimated body length between 7.2 and 7.7 m, comparable to that of the largest living male killer whales. Palynological dating of the sediment of one cranial specimen supports a Gelasian (2.6–2.1 Ma) age. This large representative of the genus *Orcinus* displays morphological features - including large, deep, and widely separated alveoli and deeply excavated condylar fossae - indicating macroraptorial feeding abilities. *Orcinus* sp. represents the largest Pleistocene delphinid yet documented and provides the earliest known evidence for the emergence of macroraptorial predation within Delphinidae, substantially predating the Middle Pleistocene divergence of modern killer whale ecotypes inferred from genomic data. By demonstrating that this feeding strategy was established more than two million years ago, our results underscore both the long-term stability of apex predation in marine ecosystems and its potential vulnerability to rapid human-induced environmental change.

## Introduction

Killer whales (*Orcinus orca*) are the largest members of the oceanic dolphin family (Delphinidae), occupy the highest trophic level in marine food webs and have few natural threats as adults. Their diet includes a wide range of marine animals, ranging from molluscs, fish, seabirds, sea turtles, to other marine mammals, including pinnipeds, toothed whales and even baleen whales (*e.g.*, Berta & Lanzetti, 2020; Ford et al., 2010). Despite this extensive menu, the many globally distributed different populations show a strong prey preference, boosting ecological specialization which is reflected by their morphological characteristics, social structure and distributional patterns (*e.g.*, De Bruyn, Tosh & Terauds, 2013; Ford, 2018). Some of these populations have been recently proposed as distinct species (Morin et al., 2024), considered subspecies by the taxonomy Committee of the Society for Marine Mammalogy: *O. orca ater* and *O. orca rectipinnus* (Committee on Taxonomy, 2026).

Killer whales are highly social animals living and hunting almost exclusively in pods, displaying a wide range of feeding strategies. Populations with a macropredatory diet deploy a grip-and-tear technique similar to some species of shark, which is a rare behaviour among toothed whales (Citron et al., 2022; Werth, 2000). Among the macropredatory ecotypes the adult males of the Antarctic Type A attain the largest body sizes, with mean lengths of approximately 7.8 m and rare individuals approaching nine m (Durban et al., 2021; Pitman, Perryman & Eilers, 2007).

Despite a burgeoning interest in the evolutionary history of killer whales, their origins remain poorly understood. This enigma largely stems from a dearth of fossils of the genus *Orcinus* and closely related extinct taxa. At present, only one extinct species of killer whale has been recognized based on reliable fossil evidence: *Orcinus citoniensis*. The holotype consists of a nearly complete skeleton of a 3.5-meter-long individual, unearthed in 1882 from a Pliocene sandstone outcrop near Cetona, Tuscany, Italy (Capellini, 1883). *O. citoniensis* is presumed to lack the macropredatory habits of its modern relatives and is inferred to have a piscivorous diet, partly because of its relatively small body size (compared to extant *Orcinus*), lower bite strength, narrower rostrum, and teeth that do not interlock. The evolution of fully developed macroraptorial traits within *Orcinus* may therefore have occurred later, possibly during the Pleistocene, in association with major environmental and faunal reorganizations (Bianucci et al., 2022; Citron et al., 2022).

The Pliocene–Pleistocene transition represents a period of profound environmental change, including global sea-level fluctuations, contraction of coastal habitats, intensified cooling and glaciation, reorganization of oceanic circulation, and the final closure of the Central American Seaway. These processes coincided with a substantial turnover in marine megafauna (Boessenecker, 2013; Pimiento et al., 2017). Pimiento et al. (2017) estimated a decline of approximately 55% in generic marine mammal diversity across this interval. Despite its pivotal role in shaping the modern marine mammal assemblage, this crucial phase of cetacean evolution remains comparatively understudied, largely because accessible Lower Pleistocene marine deposits are scarce globally (Bianucci et al., 2022).

The Natuurhistorisch Museum Rotterdam houses a rather extensive collection of 39 fossil specimens from the southern North Sea, tentatively attributed to a large *Orcinus*-like dolphin, previously unknown to science. Stratigraphic evidence indicates a Late Pliocene to Early Pleistocene age and analysis of dinoflagellate cysts corroborated an Early Pleistocene origin (as demonstrated in this work). The five most informative specimens are described here and referred to a large macroraptorial member of the *Orcinus* genus. This conclusion extends our knowledge of this peculiar feeding behaviour in delphinids, suggests that large sizes in *Orcinus* were reached much earlier than hitherto assumed (see Bianucci et al., 2022; Citron et al., 2022), and highlights resource availability and biodiversity of the Southern North Sea Basin during the Early Pleistocene.

## Material and Methods

### Specimens observed for comparison

Comparisons were made primarily with extant and fossil Globicephalinae and Orcininae (*sensu* Bianucci et al., 2022) showing skeletal elements similar in shape and/or size to those preserved in the examined North Sea specimens. Furthermore, comparisons with fossil species are limited to taxa for which the skeletal elements preserved in the examined North Sea specimens are known. Therefore, the following list contains only the taxa and related specimens that were the subject of this detailed comparison. All specimens listed below were directly observed by the authors, with the exception of the holotype of *Protoglobicephala mexicana*, for which published photographs were examined (Aguirre-Fernández et al., 2009), and three *O. orca* skulls housed in the NMNZ, for which high-resolution photographs available online were examined (https://collections.tepapa.govt.nz).

*Feresa attenuata* USNM 395177, 484995, 504916, 504917, 504918, 504919, 550389, 571268; ZMA.MAM.22687, MAM.10463; *Globicephala macrorhynchus* NMR 999000001381, 999000001443; USNM 37261, 500247, 504396, 550438, 571933; ZMA.MAM. 1908, MAM.5059, MAM.5062, MAM.8871, MAM.8872, MAM.8873, MAM.8875 MAM. 23829; *Globicephala melas* IRSNB 1514, 1514B, 1514Y; MSNUP C-302; NMR 999000001380, 999000003005, 999000003006; NMNZ MM001604, MM001622, MM001848; ZMA.MAM.11583, MAM.8881; RMNH.MAM.36395; STME B2-377, B2-1717, B2-0012, B2-1213, A.V.2001; *Grampus griseus* MSNUP C-294, C-295, C-296, C-297; USNM 572313, 572921; NMNZ MM002856; USNM 550391, 571006, 571349; ZMA.MAM.7793, MAM.7794, MAM.20316, MAM.147.010; *Hemisyntrachelus cortesii* NMR 999100008180 (cast); *Hemisyntrachelus oligodon* SMNK-PAL.3841; *Hemisyntrachelus pisanus* NMR 999100008159; *Hemisyntrachelus* sp. NMR 999100016974; *Orcinus citoniensis* MGGC 8560, 8563–8565; *Orcinus orca* IRSNB.1511; MSNUP C-298, C-301; RMNH.MAM.17711, MAM.7514, MAM.1487, MAM.13487, MAM.31219, MAM.1982; NHMUK ZC.1917.10.2.1, ZC.1924.11.4.1, ZC.1924.11.4.2, ZC.1934.7.23.2, SW.1944/2, SW.1943/11, SW.1943/9, SW.1918/10, SW.1918/12, SW.2001/234, SW.1937/27, ZD.1857.6.12.14, SW.1927/28, ZD.1992.7, SW.1926/44, GERM.1165a, GERM.1165b, GERM.1165c, GERM.361c, ZD.1887.5.20.1; MM002615; NMR 9990000933; STME B2-09; USNM 11980, 23004; ZMA.MAM.4211, MAM.13492; *Peponocephala electra* RMNH.MAM.38439, MAM.38438, MAM.22254, MAM.38235, MAM.15956, MAM.38437; USNM 504948, 550399; ZMA.MAM.15961, 15962; *Protoglobicephala mexicana* UABCS ISJ/3008; *Pseudorca crassidens* MSNUP C-299; C-300; USNM 219325; ZMA.MAM.770, MAM.12898, MAM.21168, MAM.21186, MAM.11876, MAM.13574, MAM.13084, MAM.145260; RMNH.MAM.2393b; STME B2-717; *Rododelphis stamatiadisi* MMPS 544.

### Anatomical terminology

The anatomical terminology follows Ichishima (2016), Mead & Fordyce (2009), Roston, Boessenecker & Geisler (2023) and Telizhenko & Gol’din (2025) for the skull and Evans & De Lahunta (2013) for postcranial skeletal elements.

### Measurements and fossil preparation

Measurements were recorded with callipers. Photographs were taken with a Canon EOS 70D(W) and 60 mm zoom lens (Canon Inc., Tokyo, Japan). Images were subsequently edited and assembled in CorelDRAW Graphics Suite 2019 (Corel Corp., Ottawa, ON, Canada). Photogrammetric three-dimensional (3D) models were generated using the KIRI Engine mobile application (KIRI Innovations Ltd., Toronto, ON, Canada).

An air-compressor-powered air scribe was used to clear the posterior side of NMR999100007979 of sediments. To prevent deterioration, all specimens were prepared by desalination using a natrium-poor liquid (Dutch municipal water containing 20 mg/L natrium) and treated with Paraloid B72 dissolved in acetone (100 g in 1,000 ml, resulting in a 10% solution).

### Statistical analysis

In order to estimate the total body length based on the examined fossil remains, predictive regression equations were generated, using 12 measurements per mandible of 18 extant *O. orca* and one measurement per atlas vertebra of 15 extant killer whale specimens. Only adult individuals, of which the total body length (TBL) was known, were included in the sample. For each specimen with a complete dataset of measurements, a geometric mean (GM) was calculated. All measurements, the TBL and the GM were then log_10_ transformed, before regression equations were generated. For each equation, R^2^, percent prediction error (%PE) and percent standard error (%SEE) were calculated, to evaluate the performance of the equations. All computations and analyses were performed using a Jupyter Notebook (Tables S1–S4), running Python version 3.10.12 (Python Software Foundation, https://www.python.org/).

### 3D acquisition and digital reconstruction

The 3D textured model of the MSNUP C-301 skeleton of *Orcinus orca* was generated using a SHINING EinScan Pro HD (version 3.7.0.3; SHINING 3D Tech Co., Ltd, Hangzhou, China) structured-light scanner (Merella et al., 2023).

The 3D textured models of the *Orcinus* sp. cranial remains (NMR 07979 and NMR 07974) were produced using photogrammetry from smartphone-acquired images processed with Kiri Engine (KIRI Innovations Ltd.).

To estimate total body length, the fossil cranial models were digitally aligned and superimposed onto the corresponding anatomical regions of the MSNUP C-301 skeletal model using Blender 3.0.1 (Blender Foundation). Scaling was performed based on shared anatomical landmarks to ensure proportional correspondence between models. The estimated cranial dimensions were subsequently used to extrapolate total skeletal length by reference to the complete MSNUP C-301 skeleton. Final graphical outputs were prepared using CorelDRAW Graphics Suite 2019 (version 19).

### Palynological preparation and analysis

The age-interpretation of this study relies on the analyses of organic-walled dinoflagellate cysts (dinocysts) retrieved from the attached sedimentary material of a partial skull of *Orcinus* sp. (NMR999100007979). Dinocysts are resistant capsules (cysts), which are, as part of a complex life-cycle, produced by dinoflagellates, a group of unicellular eukaryotic algae. They are morphologically diverse and distinct, which enable the taxonomy of this microfossil group to be very well-resolved (Fensome, Williams & MacRae, 2019). Dinocysts are typically abundant in shallow (to open) marine, siliciclastic dominated depositional settings such as those of the Neogene and Early Pleistocene in northwestern Europe (*e.g.*, De Schepper & Head, 2008; De Schepper & Head, 2009). The isolation of dinocysts requires a laboratory treatment involving the use of acids to dissolve mineral components (silicates and carbonates) and subsequent sieving.

The present sample was processed for palynology by Nico Janssen (TNO, Delft, Netherlands) using the facilities of Utrecht University and Geological Survey of the Netherlands. During standard chemical processing, 35% hydrochloric acid and 30% hydrofluoric acid were used for carbonate and silicate digestion respectively (Janssen & Dammers, 2008). The resultant kerogens were sieved over a 10 µm mesh. The microscopic slides were mounted in glycerin-jelly and sealed with a cover slip.

The microscopy analysis was carried out according to standard procedures. The palynomorph association on the slides is counted, using a Leica microscope magnification of 500x (Leica Camera AG, Wetzlar, Germany), until a total of ca. 200 sporomorphs (spores and pollen) and dinoflagellate cysts is reached. The miscellaneous categories (fresh to brackish algae like *Botryococcus,* inner chambers of marine foraminifers, green algae like Tasmanaceae, *etc*.) were also calculated, but kept outside the total sum of palynomorphs. The remainder of the slide was thereafter scanned for any (rare) dinocyst species which are noted as “present” (+ symbol on the distribution chart). Diagnostic species are discussed below, a complete distribution chart including all species found is given in Fig. S3.

For the dinoflagellate cyst taxonomy, the so-called “Lentin and Williams Index” is followed (Fensome, Williams & MacRae, 2019).

The age interpretation is based on the “Last Occurrence Datum” (LOD; extinction) and “First Occurrence Datum” (FOD; origination) of dinoflagellate cysts, which are calibrated to the geological time scale (Gradstein et al., 2020). Palynological interpretation is based on references concerning the palynostratigraphy of Late Pliocene-Early Pleistocene from the southern North Sea Basin (such as: Dearing Crampton-Flood et al., 2020; De Schepper & Head, 2008; De Schepper & Head, 2009; De Schepper, Head & Louwye, 2009; Donders et al., 2018; Everaert et al., 2023; Head, 1998; Hennissen et al., 2015; Hennissen et al., 2017; Kuhlmann et al., 2006; Meijer et al., 2006; Noorbergen et al., 2015).

## Systematic Paleontology

**Table:** 

Order Cetacea Brisson, 1762
Suborder Odontoceti Flower, 1867
Infraorder Delphinida Muizon, 1988
Superfamily Delphinoidea Gray, 1821
Family Delphinidae Gray, 1821
Genus *Orcinus* Fitzinger, 1860
*Orcinus* sp.

### Referred specimens

NMR 999100007974 - posterodorsal portion of cranium, NMR 999100007979 - posterolateral left portion of cranium, NMR 999100007973 - anterior portion of left mandible, NMR 999100007982 - atlas and axis vertebrae, NMR 999100011653 - atlas and axis vertebrae, hereafter referred to as NMR 07974, NMR 07979, NMR 07973, NMR 07982, and NMR 11653, respectively.

### Locality and horizon information

The fossil specimens described herein were recovered as bycatch from commercial fishing trawlers operating in the southern North Sea, primarily between the coasts of the Netherlands and the British Isles (Fig. 1). The material originates from two offshore localities, referred to as the “Noordhinder” and “Outer Gabbard” sites, situated approximately 100 km west of Rotterdam (see also Funnell, 1996; Mol & Bakker, 2022). At both localities, sediments of the Westkapelle Ground Formation are exposed at the seafloor and have been dated to the Early Pleistocene interval (Busschers et al., 2025; Post & Bosselaers, 2005; Post & Kompanje, 2010; Post & Reumer, 2016).

**Figure 1:**
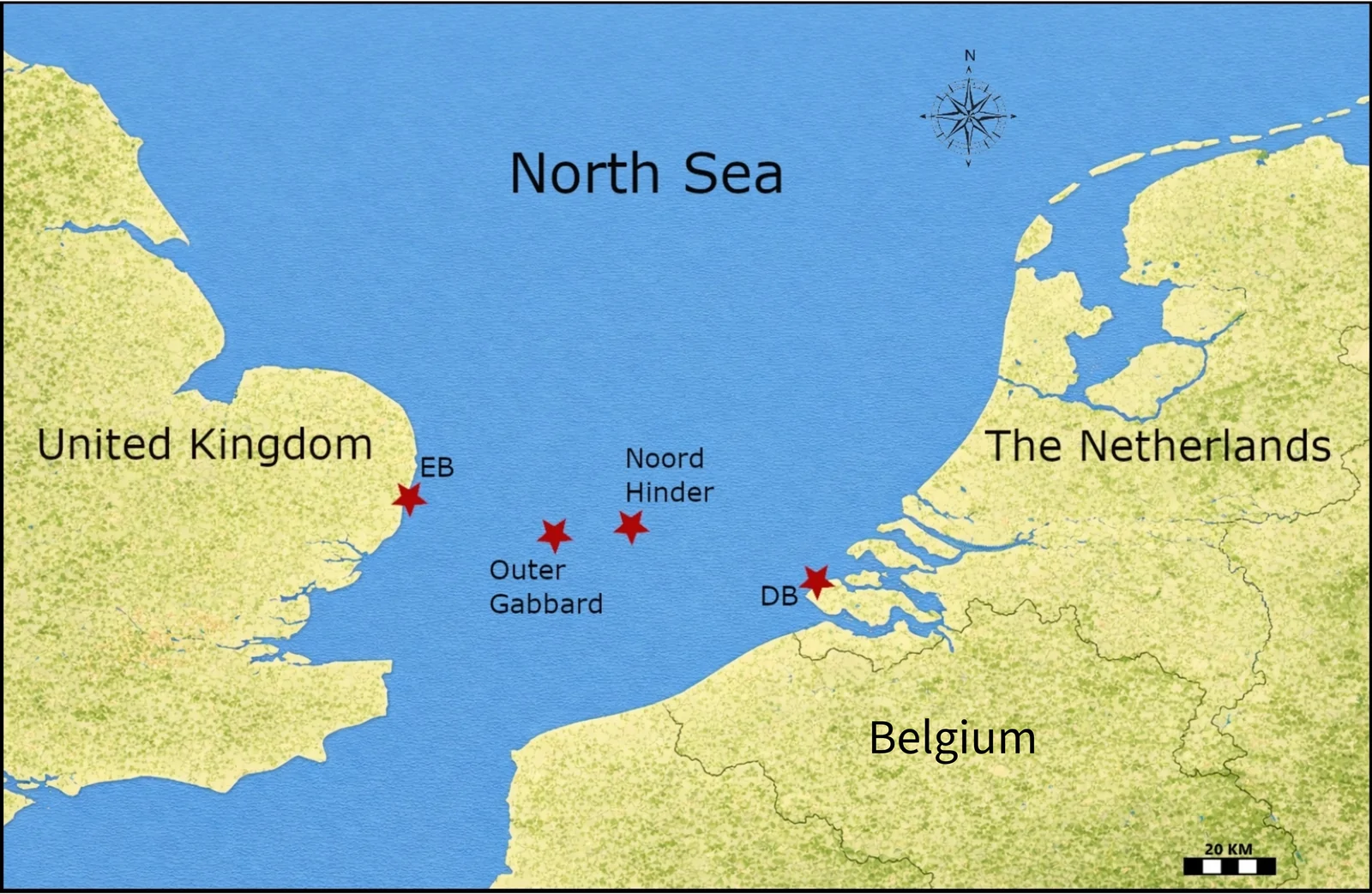
North Sea map with localities (red stars) of Late Pliocene/Early Pleistocene sites. EB, Easton Bavents cliffs, Suffolk County, East Anglia, United Kingdom; DB, Domburg, Zeeland, The Netherlands.

Fossils recovered from these deposits are rare, fully mineralized, and lack recoverable ancient DNA, in marked contrast to the more abundant and often better-preserved Late Pleistocene marine mammal remains known from elsewhere in the southern bight of the North Sea (Post, 2022). The assemblage(s) represented by the Noordhinder and Outer Gabbard material is therefore distinct and corresponds to a poorly understood marine mammal fauna or faunae of Early Pleistocene age (Post & Bosselaers, 2005). The assemblage(s) include taxa such as the walrus *Ontocetus*, and at least two oceanic delphinids, *Platalearostrum hoekmani*, and a representative of the genus *Hemisyntrachelus* Brandt, 1873 (Post & Kompanje, 2010; Bakker & Post, 2020; Boisville, Chatar & Kohno, 2024). Comparable occurrences in the broader region and with a better constrained stratigraphical context include *Ontocetus* from the Norwich Crag Formation 2.2–1.7 Ma at Easton Bavents, United Kingdom, and material referable to *Ontocetus* and *Hemisyntrachelus* from Domburg, the Netherlands, originating from the marine Maassluis Formation 2.0–1.8 Ma (Boisville, Chatar & Kohno, 2024; Holt-Wilson, 2015; Preece et al., 2020; Post & Bosselaers, 2017; Scager et al., 2017; Wesselingh et al., 2025). Recent evidence suggests that both occurrences may in fact be slightly older, approximately 2.2–2.4 Ma (Wesselingh, pers. comm., 4 February 2026). A more precise stratigraphic placement beyond the Late Pliocene–Early Pleistocene interval of the specimens here examined, as well as various previously described taxa was hampered, owing to the impossibility to directly examine the Noordhinder and Outer Gabbard outcrops situated along the seafloor, and the subsequently ex-situ context of the fossil material. However, the palynological analysis of the attached sedimentary material of NMR07979 -a posterolateral portion of the skull- indicates an age of 2.6–2.1 Ma, corresponding to the earliest Pleistocene (Gelasian) (see paragraph on palynological analyses below)

### NMR 07974–Posterodorsal portion of cranium

#### Preserved bones

NMR 07974 (Fig. 2) consists of a fragmentary cranium preserving the vertex and adjacent regions. The preserved elements include the interparietal and portions of the cribriform plate, frontals, right maxilla and supraoccipital.

**Figure 2:**
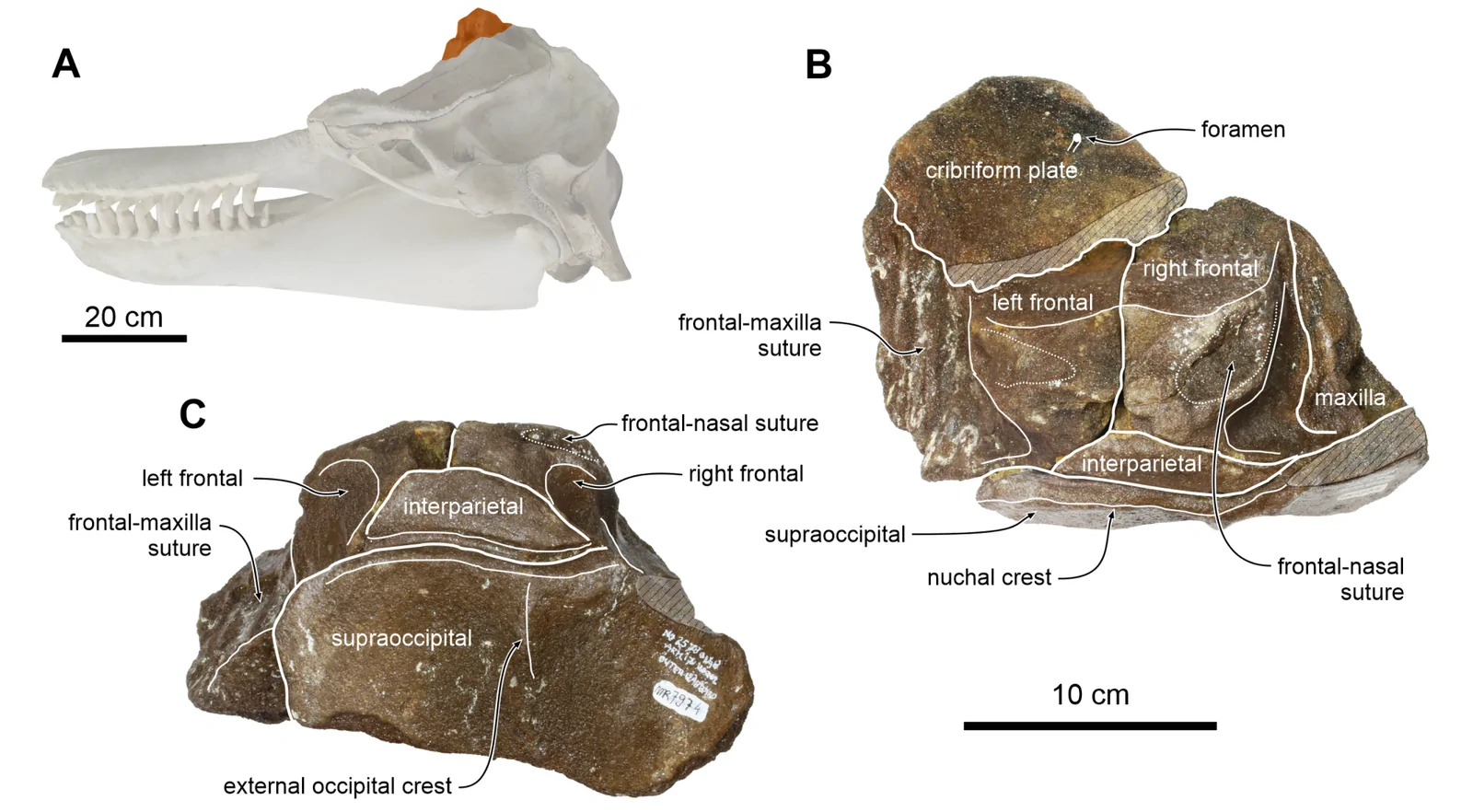
NMR 07974 *Orcinus* sp., posterodorsal portion of cranium. (A) Skull of extant *Orcinus orca* (MSNUP C-301) in left lateral view indicating the position of the preserved bones (orange); (B) dorsal view; (C) posterior view. Linear hatching indicates major breaks.DOI of 3D-scan: https://doi.org/10.17602/M2/M842064.

#### Ontogeny

The large interparietal suggests that NMR 07974 could be a young animal, since we observed small or absent exposure at the vertex of this bone in the adult crania of *O. orca*, the species to which NMR 07974 shows the greatest similarities (see comparison below). The incomplete closure of the sutures and the missing of the posteromedial portion of the maxilla further supports this interpretation.

#### Description

In dorsal view, the anteriormost preserved area consists of a slab of highly weathered spongy bone referable to the cribriform plate, overlying the anteromedial portions of the robust frontals. Based on the flat surface of the anteromedial portion of the frontals immediately posterior to the broken posterior margin of the cribriform plate, it is likely that the cribriform plate originally extended further posteriorly, forming a broad plate, weakly bent posterodorsally and covering the frontals up to their sutures with the nasals. The cribriform plate is pierced by a small foramen (three mm in diameter) located close to the midline of the skull.

The frontals, whose dorsomedial suture is clearly visible, form a trapezoidal, prominent structure at the vertex, 10.7 cm wide. Although their dorsal surface at the vertex is partially eroded, traces of the sutures with the missing nasals are still well discernible. Notably, an oval impression identified as the nasal suture, measuring 4.2 × 1.6 cm is present on the right frontal.

Laterally to the right side of the vertex, a small preserved posteromedial fragment of the right maxilla partially overlaps the right frontal, whereas laterally on the left side, the dorsal surface of the left frontal bears deep grooves corresponding to the sutural contact with the missing left maxilla.

The posterior portion of the vertex shows a strong lateral constriction, and its dorsal surface is occupied by a large, triangular interparietal. The right side of the interparietal extends 1.5 cm farther laterally than the left relative to the external occipital crest, imparting a distinctly asymmetrical appearance to this region.

The nuchal crest is robust and sinusoidal in outline, and the supraoccipital slopes abruptly posteroventrally.

In posterior view, a prominent external occipital crest extends vertically across the supraoccipital. Relative to this crest, the frontal median suture is displaced 3.5 cm to the left, further emphasizing the cranial asymmetry. At the level of this crest, the nuchal crest exhibits a weak posterior convexity.

Ventrally the cranial vault is exposed consisting of paired globular impressions separated by a marked ridge (the internal occipital crest), representing the imprint of the cerebral hemispheres. On close inspection, numerous small sulci are visible.

#### Comparison

NMR 07974 can be referred to Delphinidae based on a broad, flat cribriform plate, not divided in two by a crest and that does not rise abruptly posterodorsally (Muizon, 1988; Bianucci, 2013). Within Delphinidae, the morphology of the vertex region of NMR 07974 closely matches that of *Orcinus orca* and shares with this species the following combination of characters: (1) very large overall size (vertex width 10.7 cm), exceeding that of most delphinids; (2) the presence of relatively small sutural excavations on the frontals, indicating nodular nasals that were weakly sutured to the frontals (a condition commonly observed in *O. orca*, in which the nasals are frequently lost in museum specimens but leave comparable sutural traces); and (3) a sinusoidal nuchal crest exhibiting a posterior convexity at the level of the external occipital crest, flanked by posterior concavities on either side.

NMR 07974 differs from adults of extant *O. orca* primarily in the broader exposure of the interparietal at the vertex. As discussed above, this feature may be related to incomplete ontogenetic maturity rather than representing a taxonomically significant distinction.

A similar sinusoidal nuchal crest morphology and occasional loss of nasals are also observed in *Pseudorca crassidens*, which nevertheless differs distinctly from NMR 07974 in possessing a vertex that is more anteroposteriorly elongated and laterally compressed, as well as nasals that descend anterolaterally onto the vertex.

*Globicephala* spp. differ from NMR 07974 in having large, globose nasals that are firmly sutured to the frontals and cover most of the dorsal surface of the vertex in adult individuals, as well as an arched and posteriorly convex nuchal crest. Similarly enlarged and firmly sutured nasals are also present in *Grampus griseus*, which is further distinguished by an S-shaped nuchal crest, with its left half positioned more anteriorly than the right.

The vertex regions of *Feresa attenuata* and *Peponocephala electra*, in addition to being substantially smaller than that of NMR 07974, display a sinusoidal nuchal crest that is inverted relative to the condition observed in *O. orca* and NMR 07974, exhibiting a posterior rather than anterior concavity at the level of the external occipital crest. Moreover, in *F. attenuata*, the vertex rises less steeply posterodorsally than in NMR 07974 and *O. orca*, and the nasals are rectangular with their respective principal axes oriented orthogonally to the sagittal plane. In *P. electra*, the vertex is further characterized by an extremely short anteroposterior length.

The cranium of *Orcinus citoniensis* from the Pliocene of Italy - the only extinct species of *Orcinus* based on diagnostic material - is significantly smaller than both *O. orca* and NMR 07974. Unfortunately, the vertex of the holotype and only known specimen is fragmentarily preserved, precluding detailed comparison with NMR 07974; however, the right nasal is preserved and is relatively small but firmly sutured to the frontal (Citron et al., 2022).

Among other extinct delphinids, *Hemisyntrachelus cortesii* from the Pliocene of Italy shows some affinity with NMR 07974 in nasal morphology (Bianucci, 1996; Bianucci, 1997). Indeed, examination of casts of the holotype (the original specimen being lost) indicates small, nodular nasals. In referred specimens, the nasals are missing and the frontals show small sutural excavations similar to those observed in NMR 07974 and *O. orca*. Nevertheless, the vertex of *H. cortesii* differs clearly in its smaller size, lack of a marked postnasal constriction, and straight nuchal crest in dorsal view.

The vertex of *Rododelphis stamatiadisi* from the Pleistocene of Greece differs markedly from NMR 07974, resembling *P. crassidens* in its pronounced anteroposterior elongation, lateral compression, and nasal size and shape (Bianucci et al., 2022).

Finally, *Protoglobicephala mexicana* from the Pliocene of Mexico exhibits a vertex closely resembling that of *Globicephala* spp., characterized by very large nasal sutural excavations on the frontals and an arched, posteriorly convex nuchal crest (Aguirre-Fernández et al., 2009).

### NMR 07979–Posterolateral left portion of cranium

#### Preserved bones

NMR 07979 (Fig. 3) consists of the left squamosal and associated portions of parietal, alisphenoid, basisphenoid, basioccipital and exoccipital.

**Figure 3:**
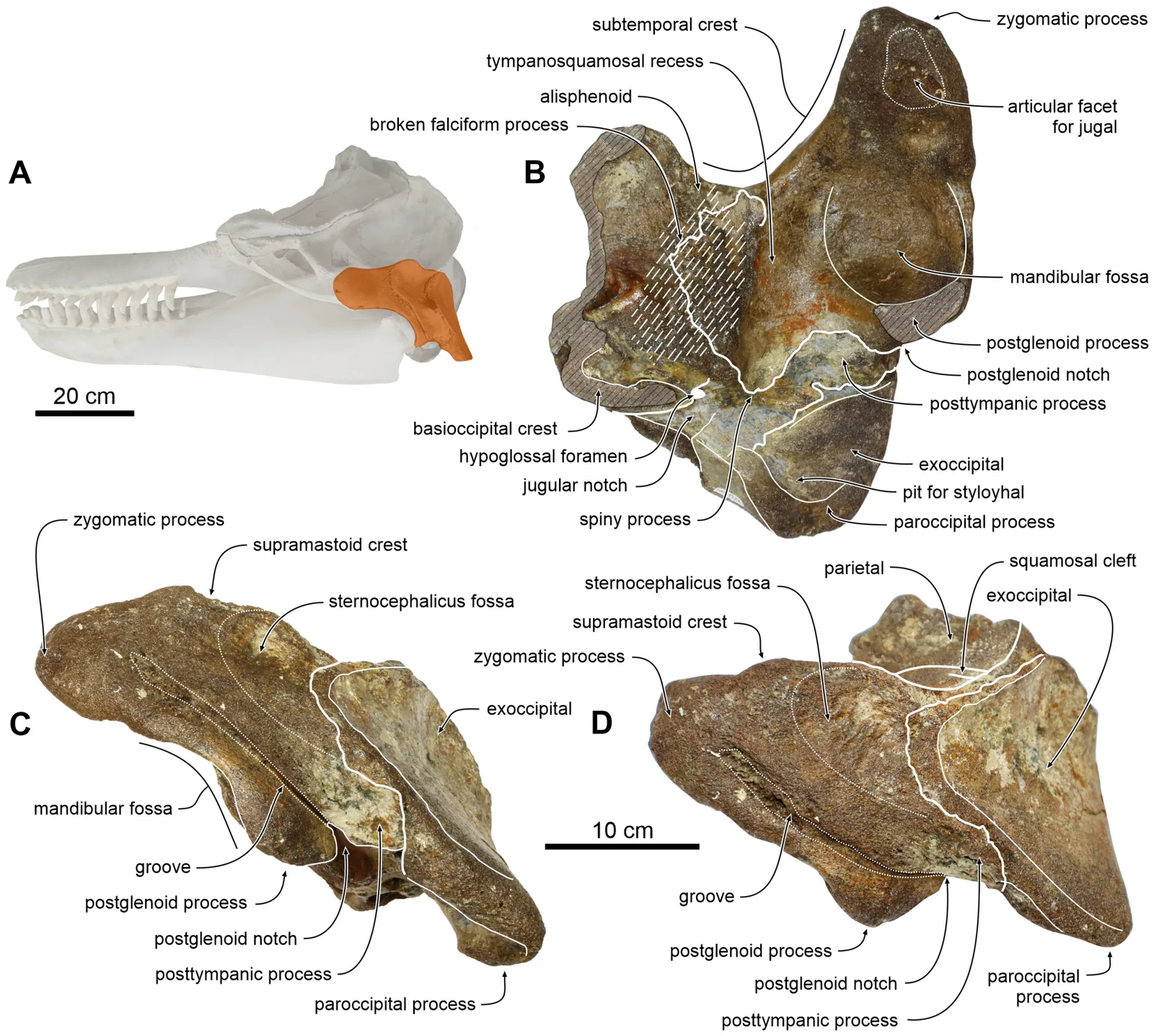
NMR 07979 *Orcinus* sp., posterolateral left portion of cranium. (A) Skull of extant *Orcinus orca* (MSNUP C-301) in left lateral view indicating the position of the preserved bones (orange); (B) ventral view; (C) lateroventral view; (D) lateral view. Linear hatching indicates major breaks, dotted hatching eroded surfaces.DOI of 3D scan: https://doi.org/10.17602/M2/M842056.

#### Ontogeny

NMR 07979 is attributed to an adult individual because of the visible but closed cranial sutures.

#### Description

In lateral view, the zygomatic process of the squamosal is anteroposteriorly short, robust and roughly square in outline. Its dorsal margin is defined by a well-developed supramastoid crest, the posterior half of which is distinctly dorsally convex. Medial to the supramastoid crest, the squamosal plate exhibits a squamosal cleft near the squamosal–parietal suture. This narrow fissure has been reported in the squamosal of some mysticetes and a few odontocetes, and among delphinids it is known only in *O. orca* (Telizhenko & Gol’din, 2025). Just ventral to the supramastoid crest, a wide and anteroposteriorly elongated sternocephalicus fossa extends anteriorly to a distance of seven cm from the anterior apex of the zygomatic process. Posterior to the sternocephalicus fossa, an anteriorly convex suture separates the squamosal from the exoccipital.

An oblique groove is clearly discernible anteroventrally to the sternocephalicus fossa. It originates five cm from the apex of the zygomatic process, extends posteroventrally, and ends posteriorly in the postglenoid notch. Posterior to the short but robust postglenoid process and the posttympanic processes, the paroccipital process of the exoccipital represents the ventralmost extent of this cranium fragment.

In ventral view, a large (transverse diameter 2.1 cm), pear-shaped articular facet for the missing jugal is present on the anterior portion of the zygomatic process. The posterior half of the ventral surface of the zygomatic process is occupied by a large (transverse diameter 7.6 cm), relatively deep, circular, and predominantly ventrally orientated mandibular fossa, posterolaterally bordered by a prominent, though partially eroded, postglenoid process. Although the fragmentary nature of the specimen makes precise orientation difficult, superimposition of the 3D model of the preserved cranial fragment onto 3D models of complete skulls of *O. orca* and other delphinids suggests that the main axis of the zygomatic process, as well as its lateral margin, were approximately parallel to the sagittal plane of the skull in ventral view. The anteromedial margin of the zygomatic process forms the lateral portion of a wide and smoothly curving subtemporal crest. The mandibular fossa is medially bordered by a flat to slightly concave tympanosquamosal recess.

Although the medial margin of the squamosal is damaged by erosion, from anterior to posterior the squamosal-alisphenoid suture, the broken broad falciform process, and the pointed spiny process can be distinguished. Just posterior to the postglenoid process, the posttympanic process significantly extends in an anteroposterior direction. The posterolateral portion of the ventral surface of the specimen is occupied by the protuberant paroccipital process of the exoccipital bearing a deep, oval, anteriorly directed articular facet for the stylohyoid. A deep jugular notch separates the paroccipital process from the eroded basioccipital crest. Anteromedial to the paroccipital concavity, the basioccipital is perforated by a hypoglossal foramen measuring 0.6 cm in diameter.

In medial view, the imprint of the left cerebral hemisphere is visible at the centre of the damaged bone. The anterodorsal portion consists of the parietal, whereas the anteroventral portion is formed by the alisphenoid, underlined by a strip of basisphenoid and basioccipital that form most of the ventral part of the cranial vault. The basisphenoid also accommodates the squamosal along its longitudinally directed lateral margin. The squamosal is dorsoposteriorly bounded by the exoccipital and ventroposteriorly bordered by a narrow strip of basioccipital.

The posterior view of the NMR 07979 fragmentary cranium is almost entirely occupied by the exoccipital. The prominent supramastoid crest is also visible, limiting the squamosal plate ventromedially and the temporal fossa ventrolaterally.

#### Comparison

Although the fragmentary cranium NMR 07979 does not preserve unambiguous delphinid synapomorphies, its morphological affinities with some delphinids are evident. In particular, NMR 07979 shares with *Orcinus orca* a combination of characters that are not observed together in other large delphinids with a close cranial morphology. These include: (1) very large overall size, as reflected by the distance between the sternocephalicus fossa and the postglenoid process (18.7 cm in NMR 07979, compared with 10.4 cm in the largest observed *Globicephala melas* specimen); (2) a supramastoid crest whose posterior half is markedly dorsally convex, a condition observed in most, though not all, examined *O. orca* specimens; (3) the presence of a distinct squamosal cleft on the squamosal plate; (4) a very large facet for jugal articulation on the anterior surface of the zygomatic process; and 5) a well-developed paroccipital process of the exoccipital bearing an anteriorly directed articular facet for the stylohyoid on its ventral surface.

In addition, NMR 07979 shares several other characters with *O. orca*, although these may also occur individually in other delphinids. These include a robust zygomatic process in lateral view, a deep and elongated oblique groove ventral to the broad sternocephalicus fossa, an orientation of the main axis of the zygomatic process approximately parallel to the sagittal plane of the skull in ventral view, and a deep, broad, circular, and ventrally oriented mandibular fossa.

The differences observed between NMR 07979 and extant *O. orca* are limited and include: (1) a more anteriorly extended sternocephalicus fossa, accompanied by a greater inclination of the oblique groove ventral to this fossa; and (2) a shorter zygomatic process in NMR 07979. Although post-mortem abrasion could have accentuated the apparent shortening of the zygomatic process, the well-preserved jugal articular facet suggests that the apex of the process is close to its original extent.

Among other extant delphinids, *Pseudorca crassidens* shares with NMR 07979 and *O. orca* a zygomatic process whose main axis is oriented parallel to the sagittal plane and a relatively large, ventrally oriented mandibular fossa. However, *P. crassidens* differs in having a significantly smaller facet for jugal articulation, accompanied by distinctive indentations on the anteroventral surface of the zygomatic process that are absent in both NMR 07979 and *O. orca*. Furthermore, in lateral view, the zygomatic process of *P. crassidens* is more elongated and pointed, the supramastoid crest is lower, and the oblique groove ventral to the sternocephalicus fossa is weakly developed or absent.

Some specimens of *Globicephala* spp. and *Grampus griseus* exhibit a zygomatic process that superficially resembles that of NMR 07979 in lateral view, including a comparable anterior extension of the sternocephalicus fossa. In a limited number of specimens (*e.g.*, *G. griseus*, MSNUP C.297), the oblique groove ventral to this fossa is also deep and anteriorly extended. Nevertheless, both *Globicephala* spp. and *G. griseus* differ from NMR 07979 in exhibiting an anterolaterally inclined zygomatic process in ventral view, resulting in a more anteriorly open subtemporal crest, as well as a smaller, more ventromedially oriented mandibular fossa. Additionally, *G. griseus* is characterized by a distinctive anterior indentation of the zygomatic process in lateral view.

An anterior inclination of the zygomatic process and a small, more ventromedially oriented mandibular fossa are also present in *Feresa attenuata* and *Peponocephala electra*. These taxa further differ from NMR 07979 in possessing a more gracile and anterodorsally inclined zygomatic process, a less developed supramastoid crest, and a short or absent oblique groove ventral to the sternocephalicus fossa.

Comparison between NMR 07979 and *Orcinus citoniensis* is complicated because the posterolateral portion of the holotype cranium of the latter is obscured by sediment and by the articulated right mandible. Moreover, the zygomatic process of *O. citoniensis* has been affected by fractures and related diagenetic compression and therefore does not appear correctly oriented when the skull is placed in lateral view (Citron et al., 2022, fig. 3). However, direct observations indicate that the zygomatic process of the holotype has the characteristic robust and square outline seen in *O. orca*. It is probably shorter than *O. orca* and, for this character, more similar to NMR 07979, although markedly smaller overall.

The posteroventral portion of the skull of *Hemisyntrachelus cortesii*, preserved in the holotype and in two referred specimens (Bianucci, 1996; Bianucci, 1997), shows a zygomatic process that is significantly more gracile and elongated than that of NMR 07979.

The holotype of *Rododelphis stamatiadisi* preserves only the facial bones of its neurocranium and the isolated left squamosal that is too abraded to be compared with NMR 07979 (Bianucci et al., 2022, fig. S1). No basicranial bones are preserved in the holotype of *Protoglobicephala mexicana* preventing comparison with NMR 07979 (Aguirre-Fernández et al., 2009).

### NMR 07973–Anterior portion of left mandible

#### Preserved bone

NMR 07973 (Fig. 4) consists of the anterior portion of a left mandible, with a total of eight alveoli preserved and including most of the symphyseal region. The apex and most of the lateral wall are missing.

**Figure 4:**
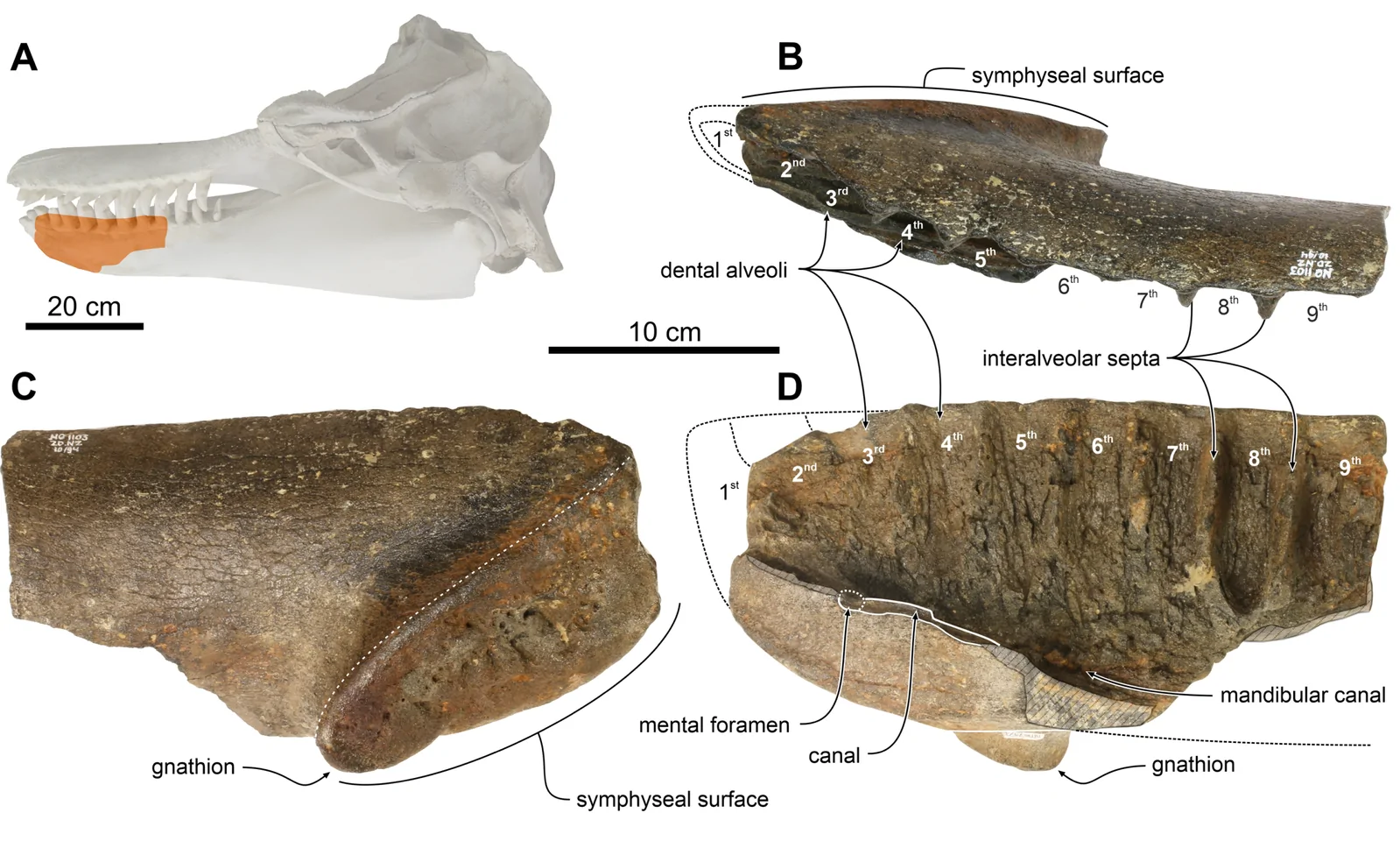
NMR 07973 *Orcinus.* sp., anterior portion of the left mandible. (A) Skull of extant *Orcinus orca* (MSNUP C-301) in left lateral view reporting the preserved bone (orange); (B) dorsal view; (C) medial view; (D) lateral view.

#### Ontogeny

The robustness of the bone and the well-defined alveoli suggest a mature individual, a hypothesis further supported by the presence of a very ventrally prominent gnathion, a character observed only in a mandible of a 62-year-old extant *O. orca* (Fung, 2016; fig. 42A).

#### Description

In dorsal view, the preserved medial halves of the second to the ninth alveoli are visible, whereas only the posterior margin of the first alveolus is preserved. The first five alveoli (1st–5th) are located on the symphyseal portion of the mandible. The anteroposterior diameter of the alveoli is approximately three cm, and adjacent alveoli are separated by interalveolar septa with preserved portions showing a minimum length of 0.5 cm. Near the posterior end of the symphysis, the medial margin of the mandibular ramus bends abruptly posteromedially, suggesting a rounded lateral outline of the articulated mandibles in dorsal or ventral view. Owing to the fragmentary nature of the specimen and the fact that the symphyseal surface is not completely flat, the symphyseal angle cannot be estimated precisely, but it approached 30°.

In lateral view, the absence of most of the lateral wall allows observation of the considerable depth of the alveoli, their rectilinear shape and, for the fourth to ninth alveoli, their vertical orientation. The eighth alveolus, the best preserved and the only one retaining its ventral wall, reaches a depth of 9.2 cm. The mediolateral width of the preserved portion of the posterior septum of this alveolus is 2.5 cm. Although this measurement is incomplete owing to the loss of a substantial part of the lateral side of the mandible, its general outline supports the interpretation that the alveoli were anteroposteriorly compressed. Indeed, given the likely significant original transverse width of the mandible, the mediolateral diameter of the alveolus can be estimated at approximately four cm, significantly exceeding its anteroposterior diameter (three cm). On the preserved ventral portion of the lateral wall, approximately five cm posterior to the preserved anterior end of the mandible, a partially preserved mental foramen is present. This foramen is connected to an internal canal, exposed by breakage, which runs posteriorly parallel to the ventral margin of the mandible.

In medial view, the incomplete, rugose symphyseal surface is roughly oval and anteroposteriorly elongated, with a longest diameter of 20 cm and a perpendicular oblique height (from anteroventral to the most posterodorsal margin) of 8.4 cm (see Table 1). Its main axis is inclined posteroventrally relative to the dorsal margin of the mandible, forming an angle of approximately 40° . The posteroventral extremity of the symphyseal surface is the ventralmost point of the mandible forming a distinct posterior protuberance corresponding to the most ventral point of the mandible (gnathion), which is clearly visible in both medial and lateral views. Immediately posterior to the symphysis, the height of the mandible measures 14.1 cm; further posterior measurements are precluded by breakage.

**Table 1:** Measurements of the mandible of extant *O. orca* and *Orcinus* sp. (NMR 07973) in centimeters.

	LS	WS	aLS	HM	A	mT	ML	TBL
NHMUK ZC. 1917.10.2.1	20.6	9.5	20.1	13.7	25.4	3.2	95.8	777
NHMUK ZC. 1924.11.4.1	20.1	9.4	17.5	12.7	27.2	3.16	93.2	–
NHMUK ZC. 1934.7.23.2	20.5	8.7	18.4	15.2	27.6	3.14	98.6	750
NHMUK SW. 1944/2	18.3	10.5	17.8	11.6	25.3	2.86	94.2	792
NHMUK SW. 1943/11	16.2	7.6	15.7	8.8	21.2	2.46	74.2	625
NHMUK SW. 1943/9	16.5	6.7	16.1	8.3	21.3	2.34	73.4	640
NHMUK SW. 1918/10	15.8	6.8	15.9	10.5	20.2	2.14	73.0	616
NHMUK SW. 1918/12	16.4	6.6	14.3	9.4	19.2	2.17	69.7	610
NHMUK SW. 2001/234	16.4	6.3	15.4	9.2	19.7	2.27	75.2	590
NHMUK SW. 1937/27	14^ˆ^	8.1^ˆ^	12.8^ˆ^	9.5	20.8	2.21	75.4	663
RMNH.MAM.17711	14.2	6.8	13.5	8.6	18.5	1.80	70.1	500
RMNH.MAM.7514	14.6	7.4	14.0	8.3	15.4	1.92	74.8	578
RMNH.MAM.1487	15.1	6.8	14.4	9.6	15.5	1.98	74.4	621
RMNH.MAM.13487	14.6	5.7	12.7	8.9	15.2	2.04	67.9	535
RMNH.MAM.31219	15.7	7.5	14.9	8.8	17.8	2.21	71.4	580
RMNH.MAM.1982	14.6	7.4	13.4	9.1	16.2	2.04	70.6	575
ZMA.MAM.4211	14.2	6.3	11.7	6.7	19.9	2.18	67.4	550
ZMA.MAM.13492	15.2	6.4	15.1	8.8	17.8	1.71	71.9	500
NMR9990000933	13.1	6.6	9.2	9.3	17.9	1.84	72.3	600
Ecomare B2-09	15.5	7.1	15.7	8.4	19.3	2.01	75.1	550
NMR 07973	20.0	8.4	19.0^*^	16.3	23.8	2.90	–	–

**Notes.**

LS, greatest length of mandibular symphysis; WS, greatest oblique height of symphysis from anteroventral to the most posterodorsal margin; aLs, anteroposterior length of symphysis; HM, dorsoventral height of mandible directly posterior of mandibular symphysis; A, distance between anterior wall of the 3th alveoli to the most posterior wall of the 9th alveoli; mT, mean of anteroposterior measurements of 3th to 9th alveoli; ML, total length of mandible; TBL, total body length measured during stranding of specimen.

* Limited information because of break surface.

ˆ Limited information because of pathological deformation.

#### Comparison

The large size and depth of the alveoli of NMR 07973, together with their rectilinear shape and the vertical orientation of the fourth to ninth alveoli, indicate the presence of large teeth with very long, straight roots. Such dental characteristics are associated with macroraptorial feeding behavior in extant *O. orca* and *P. crassidens*, as well as in several extinct macroraptorial physeteroids *e.g.*, *Acrophyseter* spp., *Livyatan melvillei*, and *Zygophyseter varolai* (Bianucci & Landini, 2006; Lambert, Bianucci & de Muizon, 2017; Lambert & Bianucci, 2019; Peri et al., 2021). However, the mandibular symphysis of NMR 07973 is proportionally much shorter than that of all known physeteroids, rendering attribution to a macroraptorial sperm whale highly unlikely. A relatively short, not ankylosed symphysis instead characterizes most delphinids, including *Orcinus*, to which NMR 07973 is here referred, also on the basis of additional diagnostic characters discussed below.

The overall dimensions of NMR 07973 fall within the range of the largest measured mandibles of extant *Orcinus orca* (NHMUK ZC.1917.10.2.1; NHMUK ZC.1924.11.4.1; NHMUK SW.1944/2; Table 1) and clearly exceed those of *Pseudorca crassidens* and all other delphinids. The alveoli of NMR 07973 are also exceptionally large, with an anteroposterior diameter of approximately three cm and root canals reaching a depth of 9.2 cm, values that exceed those observed in *P. crassidens* and other delphinids except *O. orca*. Nonetheless, the largest extant *O. orca* individuals possess even larger alveoli, with anteroposterior diameters ranging between 4 and 4.5 cm.

Additional characters shared by NMR 07973 and *O. orca* include an elongated, elliptical symphyseal surface - differing from the shorter symphyses observed in *Feresa attenuata*, *Globicephala* spp., and *Grampus griseus*, - with its main axis strongly inclined relative to the horizontal plane, as well as an elevated mandibular height at the posterior end of the symphysis.

Moreover, (1) the estimated symphyseal angle of NMR 07973 (approximately 30°) falls within the range measured in *O. orca* (27–32°) and is distinctly lower than in *F. attenuata*, *Globicephala* spp., and *G. griseus*; (2) the number of teeth in the symphyseal portion is five in NMR 07973, compared with four to six in *O. orca*; (3) the apical portion of NMR 07973, a left mandible, is straight, contrasting with the anticlockwise torsion and more laterally directed alveoli typical of left mandibles of *P. crassidens*, *Globicephala* spp., *G. griseus*, and *F. attenuata*; and (4) the estimated mediolateral width of the ninth alveolus suggests that the tooth roots of NMR 07973 were anteroposteriorly compressed, a condition observed among delphinids only in *O. orca* and the significantly smaller *Orcinus citoniensis* (Bianucci et al., 2022).

NMR 07973 differs from all directly observed mandibles of extant *O. orca* in exhibiting a pronounced, protuberant gnathion, related to the strong posteroventral inclination of the symphyseal surface. However, a similarly developed gnathion has been reported in at least one older extant *O. orca*, suggesting that this feature may fall within the range of ontogenetic variation (Fung, 2016).

Comparisons with extinct delphinids for which mandibular material has been described (see Amane, Bianucci & Zouhri, 2025) did not reveal close morphological affinities, largely because all previously documented fossil mandibles are substantially smaller and more gracile than NMR 07973.

### NMR 07982 and NMR 11653–fused atlas and axis vertebrae

#### Preserved bones

NMR 07982 and NMR 11653 both consist of a fused, large atlas and axis, forming a robust cervical complex. Fusion is complete in both specimens and does not involve subsequent posterior cervical vertebrae. The neural arches and parts of the transverse processes are partially damaged, and NMR 07982 has suffered significant abrasion.

#### Ontogeny

The fusion of the atlas and axis, with almost completely obliterated sutures, indicates that both cervical complexes belonged to mature individuals.

#### Description

In anterior view, the condylar fossae of both the atlases are large, reniform, and deeply excavated, and are bounded lateroventrally by a prominent ridge. The two fossae are only slightly inclined ventromedially with their main axes forming together an angle of 48° in NMR 11653 (Fig. 5) and approximately 45° in NMR 07982. Their medial margins are in contact with each other in their most ventral tract. The external contour of the paired condylar fossae, best preserved in NMR 11653, is approximately circular with a transverse width of 23.4 cm in NMR 11653, very close to the estimated 23 cm of NMR 07982. Medial to the articular facets, a deep V-shaped depression is present and is filled by the odontoid process of the axis, which is completely fused to the atlas in both specimens.

**Figure 5:**
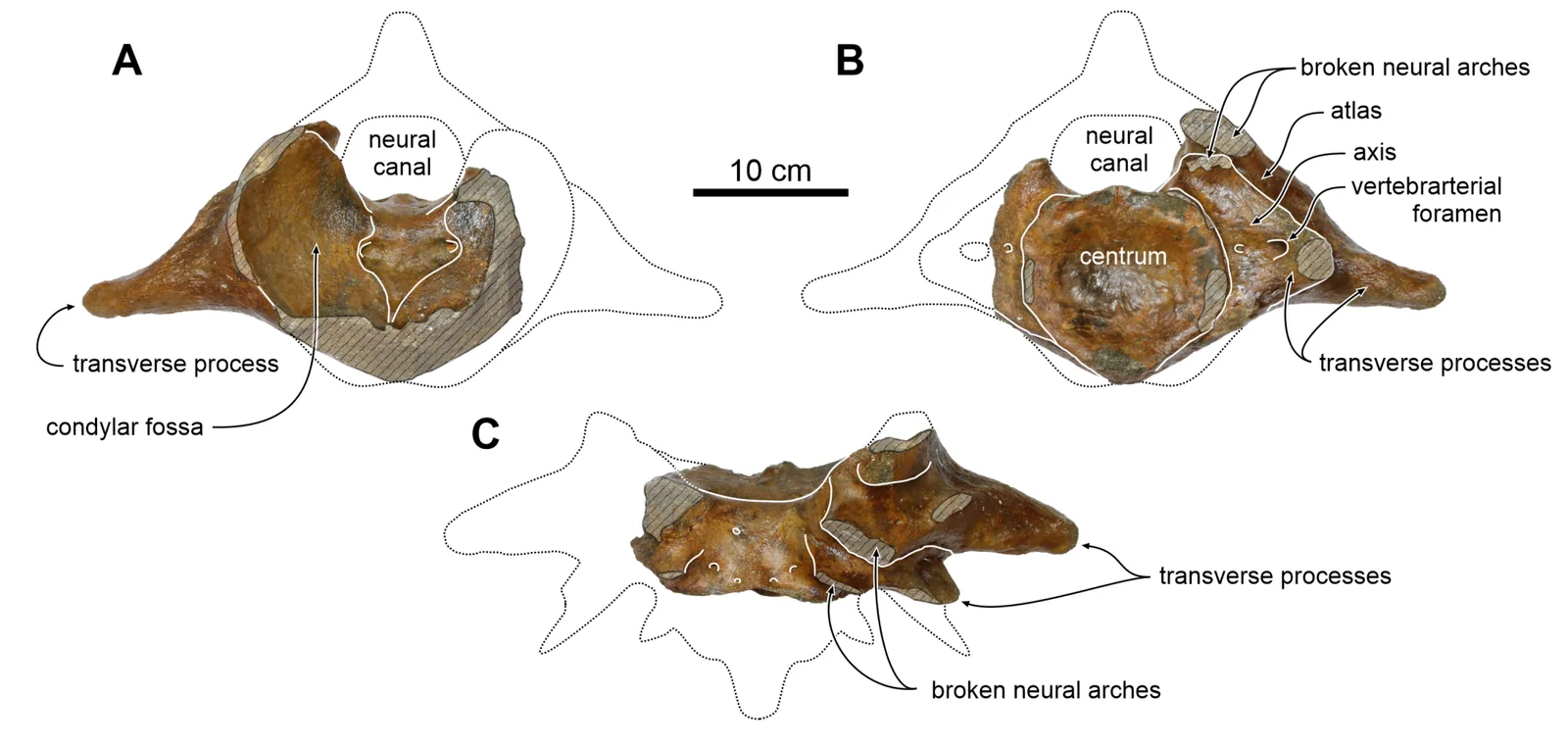
NMR 11653 *Orcinus.* sp., fused atlas and axis vertebrae. (A) Anterior view; (B) posterior view and (C) dorsal view. Linear hatching indicates major breaks.

The well-preserved left transverse process of the atlas in NMR 11653 is broad at its base and abruptly tapered distally, forming a distal styliform tip in anterior view. Its main axis bends only slightly ventrolaterally in anterior view, but distinctly posterolaterally in dorsal view. The estimated width between the transverse processes of the atlas in NMR 11653 is 41.4 cm. The left transverse process of the axis in NMR 11653 is triangular in posterior view and is similar in shape to that of the atlas, though probably originally shorter. It bends more strongly ventrolaterally than the transverse process of the atlas and is pierced by a large vertebrarterial foramen, clearly visible in posterior view.

In dorsal and ventral views, the atlas–axis suture is only partially visible and is associated with multiple small foramina in both specimens.

In posterior view, the posterior epiphysis of the axis of both specimens is circular to slightly oval and distinctly concave. The posterior epiphyseal surface is intact in both specimens and shows no evidence of fusion with subsequent cervical vertebrae. Height and width of the posterior articular surface of NMR 07982 and NMR 11653 are respectively 14.9 cm (h) and 16.1 cm (w), and 12.3 cm (h) and 13.8 cm (w).

#### Comparison

Both NMR 07982 and NMR 11653 exhibit the typical morphology of the first two cervical vertebrae of Delphinoidea, including the absence of dorsal transverse processes, a reduced or absent ventral tubercle, and marked anteroposterior compression (Muizon, 1988). In addition, fusion of at least the first two cervical vertebrae is observed in all extant delphinoids, with the exception of Monodontidae (VanBuren & Evans, 2017).

Comparisons with extant and fossil delphinoids reveal clear affinities between NMR 07982 and NMR 11653 and *Orcinus orca*, most notably in their large size, which falls within the range observed exclusively for this species (Table 2).

**Table 2:** Measurements of cervical vertebra of extant *O. orca* and *Orcinus* sp. in centimeters.

	WO	NA	WN	TBL
NHMUK ZC. 1934.7.23.2	27.2	21.3	6.7	871^*^
NHMUK ZC. 1924.11.4.1	24.7	19.5	5.4	784^*^
NHMUK ZC. 1924.11.4.2	26.7	20.0	4.4	823^*^
NHMUK ZC. 1917.10.2.1	25.5	18.0	8.1	777
RMNH.MAM.17711	16.8	19.9	9.4	500
RMNH.MAM.7514	18.2	7.2	7.1	578
RMNH.MAM.1487	18.6	9.1	8.4	621
RMNH.MAM.13487	16.6	10.9	8.8	535
RMNH.MAM.31219	16.5	8.7	7.8	580
RMNH.MAM.1982	18.1	8.1	7.8	575
ZMA.MAM.4211	17.4	10.5	7.7	550
NMR9990000933	18.4	9.6	8.3	600
Ecomare B2-09	17.0	10.2	7.2	550
MSNUP C-298	20.4	10.3	7.9	630^**^
MSNUP C-301	18.0	8.8	–	596^***^
NMR 07982	23.0	13.9	9.7	–
NMR 11653	23.4	12.8	9.6	–

**Notes.**

WO, maximum of occipital articular facets; NA, distance between ventral border of neural canal and ventral border of atlas vertebra; WN, width of neural canal measured at the base of the pedicles; TBL, total body length measured during stranding of specimen.

# Not precise because of the difficulty to measure in mounted skeleton.

– Not possible to measure in mounted skeleton.

* Calculated body length based on CBL.

** Estimated measurement based on the skeletal length of 596 mm (according to Braschi, Cagnolaro & Nicolosi, 2007) plus 55 mm of the estimate of the total length of the intervertebral disks based on Long et al. (1997, tab. 2).

*** Estimated measurement based on the skeletal length of 545 mm (according to Braschi, Cagnolaro & Nicolosi, 2007) plus 51 mm of the estimate of the total length of the intervertebral disks based on Long et al. (1997, tab. 2).

Moreover, several of the characters observed in NMR 07982 and NMR 11653 are characteristic of *O. orca*, including the reniform, strongly excavated condylar fossae, ventrolaterally bounded by a prominent ridge and only weakly inclined. In contrast, all other extant and fossil delphinids that approach *O. orca* in size, display condylar fossae that are subtriangular in shape, weakly excavated, and more steeply inclined (angle between their main axes > 52°) *e.g.*, *Pseudorca crassidens*, *Globicephala* spp., *Grampus griseus*.

The North Sea atlases further resemble *O. orca* in exhibiting medial contact between the condylar fossae, whereas in other extant and fossil delphinids the fossae are separated by a distance ranging from 1% to 12% of the external width of the condyles. Consequently, the distinct placement of the condylar fossae results in a rhomboidal external outline, in contrast to the subcircular external outline as observed in NMR 07982, NMR 11653, and *O. orca*.

Finally, both North Sea specimens share with *O. orca* the distinctive morphology of the atlas transverse process, which is abruptly tapered distally to form a styliform tip in anterior view. In other extant delphinids, the transverse process is considerably more robust, particularly in *Globicephala* spp., *F. attenuata,* and *Peponocephala electra,* whereas among fossil delphinids a similar pointed transverse process is observed only in *Hemisyntrachelus cortesi* (Bianucci, 1996). The only apparent difference between on the one side NMR 07982 and NMR 11653 and on the other side *O. orca* is the absence of ankylosis with vertebrae posterior to the axis in the former. In most examined specimens of *O. orca*, the first three cervical vertebrae were affected by ankylosis, with rarer cases showing fusion of four to five cervicals, and just a single documented instance of fusion limited to the atlas and axis (NMR999000002821, a juvenile individual from the southern North Sea). Among other extant delphinids of comparable size, marked cervical fusion is observed in *P. crassidens* (generally involving the first six cervicals), *Globicephala* spp. (first six or all seven cervicals), and *G. griseus* (all seven cervicals) (*e.g.*, Ogden et al., 1981; VanBuren & Evans, 2017). In *P. electra* and *F. attenuata*, fusion typically involves only the first three cervicals, with the centrum of the third vertebra rigidly fused to that of the axis, except in one adult specimen of *F. attenuata* (USNM 504917), in which only the atlas and axis are fused. This pattern suggests that fusion of the third cervical vertebra may be subject to intraspecific variation in delphinids. Among related fossil delphinids, the holotypes of *R. stamatiadisi* and *H. cortesi* exhibit fusion limited to the atlas and axis.

### Systematic remarks

The fragmentary nature of the available material and the limited anatomical overlap among specimens prevent direct assessment of whether all remains belong to a single species. Nevertheless, each specimen independently displays morphological features consistent with a large *Orcinus*-like delphinid, and no morphological evidence currently supports the recognition of multiple large delphinid taxa among the described material. Collectively, these observations suggest close affinities with the extant killer whale, *Orcinus orca*, or with a closely related but distinct species within the genus *Orcinus*.

The cranial material exhibits several features characteristic of *O. orca*, including nodular and weakly sutured nasals inferred from the frontal morphology; a sinusoidal nuchal crest with a posterior convexity at the level of the external occipital crest; a well-developed supramastoid crest; and the presence of a squamosal cleft. Additional diagnostic features include a short and robust zygomatic process of the squamosal, whose principal axis is interpreted as oriented approximately parallel to the sagittal plane in ventral view, a large facet for articulation with the jugal, and a deep, broad, circular, and ventrally oriented mandibular fossa.

The mandibular fragment is characterized by very large, deep, and anteroposteriorly compressed alveoli, consistent with the presence of large teeth bearing elongated, straight roots, as well as an elongated, oval, and markedly inclined symphyseal surface. Furthermore, the fused atlas-axis complexes closely resemble those of *O. orca*, notably in the presence of reniform, weakly inclined, and deeply excavated condylar fossae bounded ventrolaterally by a prominent crest, as well as the distinctive transverse process of the atlas, which tapers abruptly distally.

Most of the morphological differences observed between the North Sea specimens and extant *O. orca* can plausibly be attributed to ontogenetic variation, including the broad dorsal exposure of the interparietal at the vertex or the presence of a prominent gnathion on the mandible. Other differences, such as the apparently shortened zygomatic process, are more likely the result of post-mortem abrasion.

All preserved skeletal elements are attributed to adults, fall within the size range of large individuals of *O. orca* and are markedly larger than those of other extant and extinct delphinids, including the small-bodied *O. citoniensis* from the Italian Pliocene. Although this large body size is consistent with the morphological characters supporting attribution to *Orcinus*, size estimates for three of the four specimens examined (with NMR 07974 interpreted as an immature individual) fall within the upper quartile of published population- and measurement-based datasets for extant *O. orca* (Fig. 6). This pattern suggests that the North Sea material represents a population characterized by a greater average body size than most modern killer whale populations. However, such comparisons may be affected by a taphonomic bias, as the fossil record documents individuals that, in many cases, completed their ontogenetic trajectory and attained a large body size (Caughley, 1966; Pruden, Mendonca & Leighton, 2018). In contrast, data on living killer whales are primarily derived from *in vivo* observations encompassing broad ontogenetic and size variation. Furthermore, taphonomic selection may have favored the preservation of more robust, and therefore generally larger, individuals. In light of the ongoing debate regarding the taxonomic status of extant *Orcinus* populations that also differ in body size (Morin et al., 2024), and considering the fragmentary nature of the fossil material examined, we consider it as most prudent to refer these remains conservatively to *Orcinus* sp., pending the discovery of more complete specimens.

**Figure 6:**
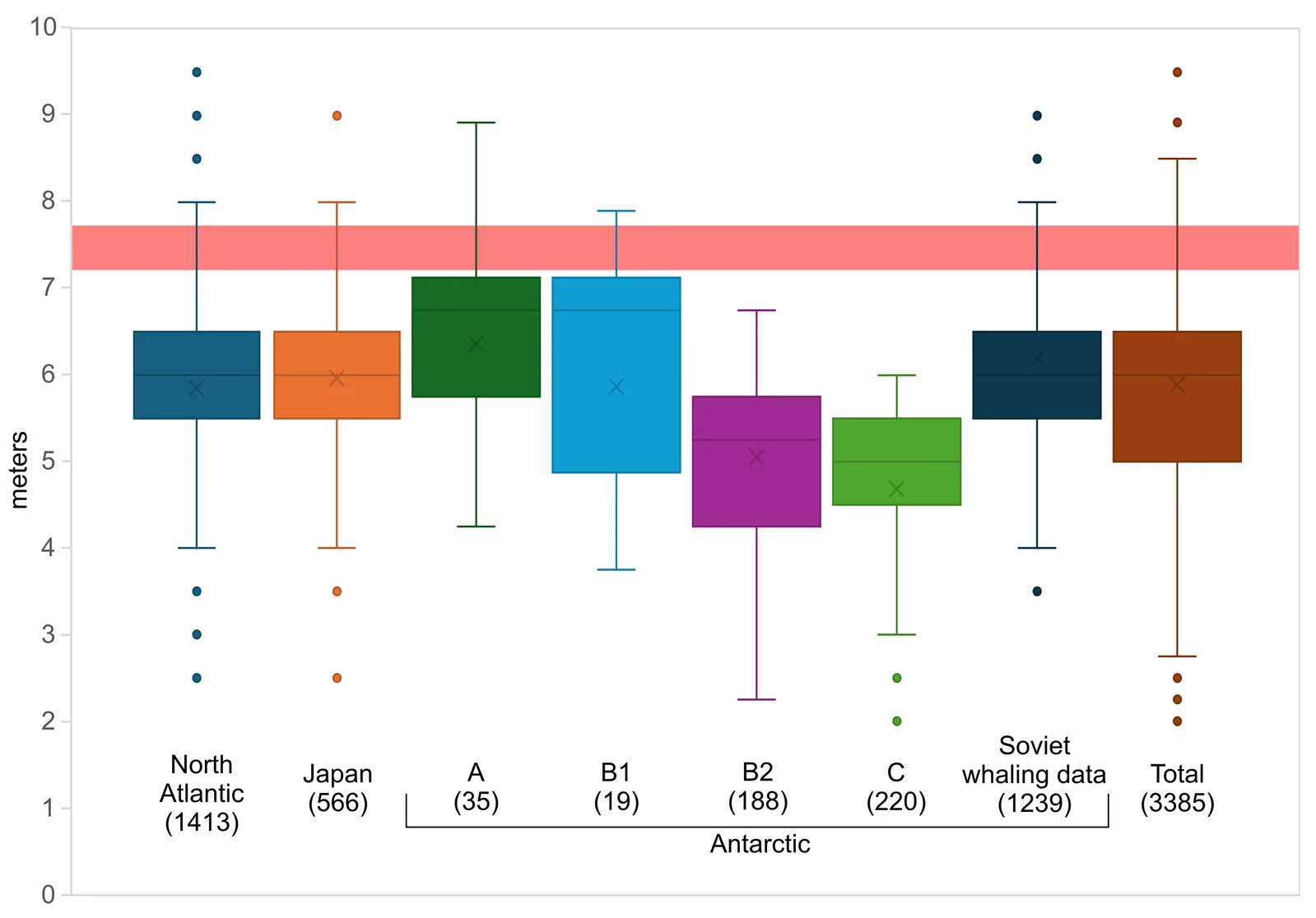
Comparison of body length in extant *Orcinus orca* ecotypes and the Gelasian *Orcinus* sp. Box-and-whisker plots showing total body length (m) of extant *Orcinus orca* from different geographic regions and ecotypes based on published datasets (sample sizes in parentheses). Boxes represent the interquartile range (IQR), the horizontal line within each box indicates the median, whiskers show the range excluding outliers, and dots represent outliers. The horizontal red bar indicates the estimated body length range (7.2–7.7 m) of the Gelasian *Orcinus* sp. from the southern North Sea Basin. The fossil estimate falls above the third quartile (Q3) of the overall distribution of extant killer whale populations. Data from Durban et al. (2021) and Pitman, Perryman & Eilers (2007).

### Body size estimation

We used two predictive regression analyses to estimate the total body length of the North Sea killer whale based on measurements of the mandible (NMR 07973) (greatest length of mandibular symphysis, greatest oblique height of symphysis from anteroventral to most posterodorsal margin, anteroposterior length of symphysis, dorsoventral height of mandible directly posterior of mandibular symphysis, distance between anterior wall of the 3th alveoli and the most posterior wall of the 9th alveoli, mean distance between the anterior wall and the posterior wall of the 3th to 9th alveoli) and of the cervical vertebrae (NMR 11653 and NMR 07982) (greatest width of condylar fossae).

In the first, we constructed an analysis using mandibular measurements of extant *O. orca* to obtain total body length in meters. The regression equation, fitted to the geometric mean of all measurements (Table S1), yielded a regression equation with an adjusted R^2^ = 0.88, %PE = 4.12, and %SEE = 0.77 (Fig. 7). Using this equation, the total body length of NMR 07973 was estimated at approximately 7.7 m (95% CI range 7.30–8.13 m; 95% PI range 6.85–8.67 m), a body length very close to the upper limit of males of some extant *O. orca* ecotypes. Indeed, males *O. orca* of the Type A ecotype from the Antarctic region are generally considered the largest killer whales (Pitman, Perryman & Eilers, 2007), with a mean body length of 7.80 m. Durban et al. (2021) encountered a male reaching 8.92 m, a length close to the upper limit of their size. However, although the length of extant killer whales is sometimes reported in the literature as long as nine m based on sightings and strandings (McClure, 2024), we found no references to lengths exceeding eight m in museum collections.

**Figure 7:**
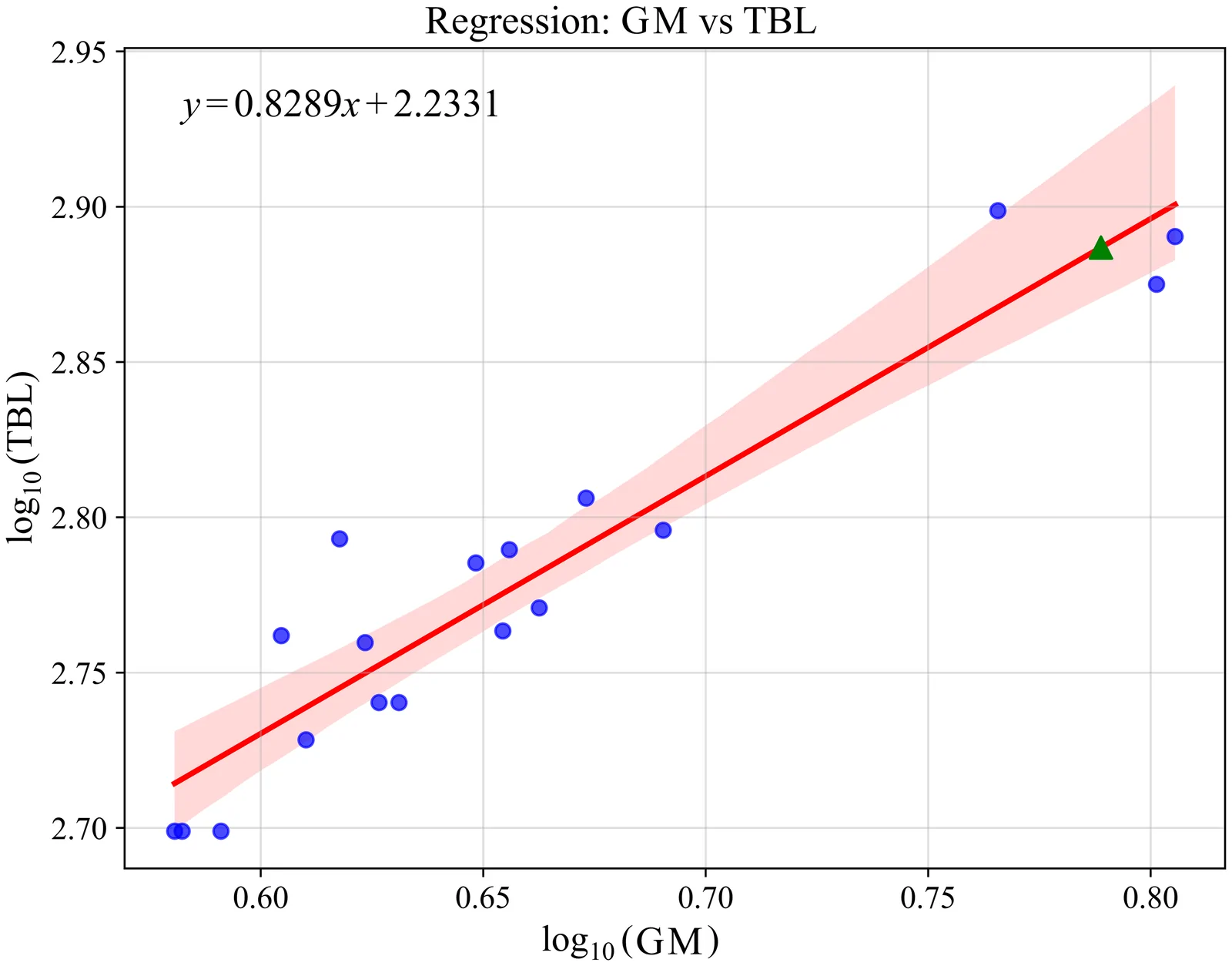
Regression analysis on mandible of *Orcinus* sp. Resulting regression of geometric mean (GM), log_10_ transformed of mandibular apex measurements (listed in Table S1) of 17 extant *Orcinus orca* and log_10_ transformed total body lengths for estimating the body length of NMR 07973, indicated with a green triangle. Blue dots represent the extant *Orcinus orca* included in this analysis.

A second regression analysis was conducted on the atlas vertebrae of *O. orca* with known body length, fitted to WO (width of condylar fossae), yielding a regression equation with an adjusted *R*^2^ = 0.95, %PE = 2.72, and %SEE = 0.6 (Fig. 8). Using this equation, the calculated body length of NMR 11653 and NMR 07982 is 7.4 m (95% CI range [7.15–7.60 m]; 95% PI range [6.74–8.06 m]) and 7.2 m (95% CI range [7.05–7.47 m]; 95% PI range [6.64–7.93 m]), respectively (Table S2).

**Figure 8:**
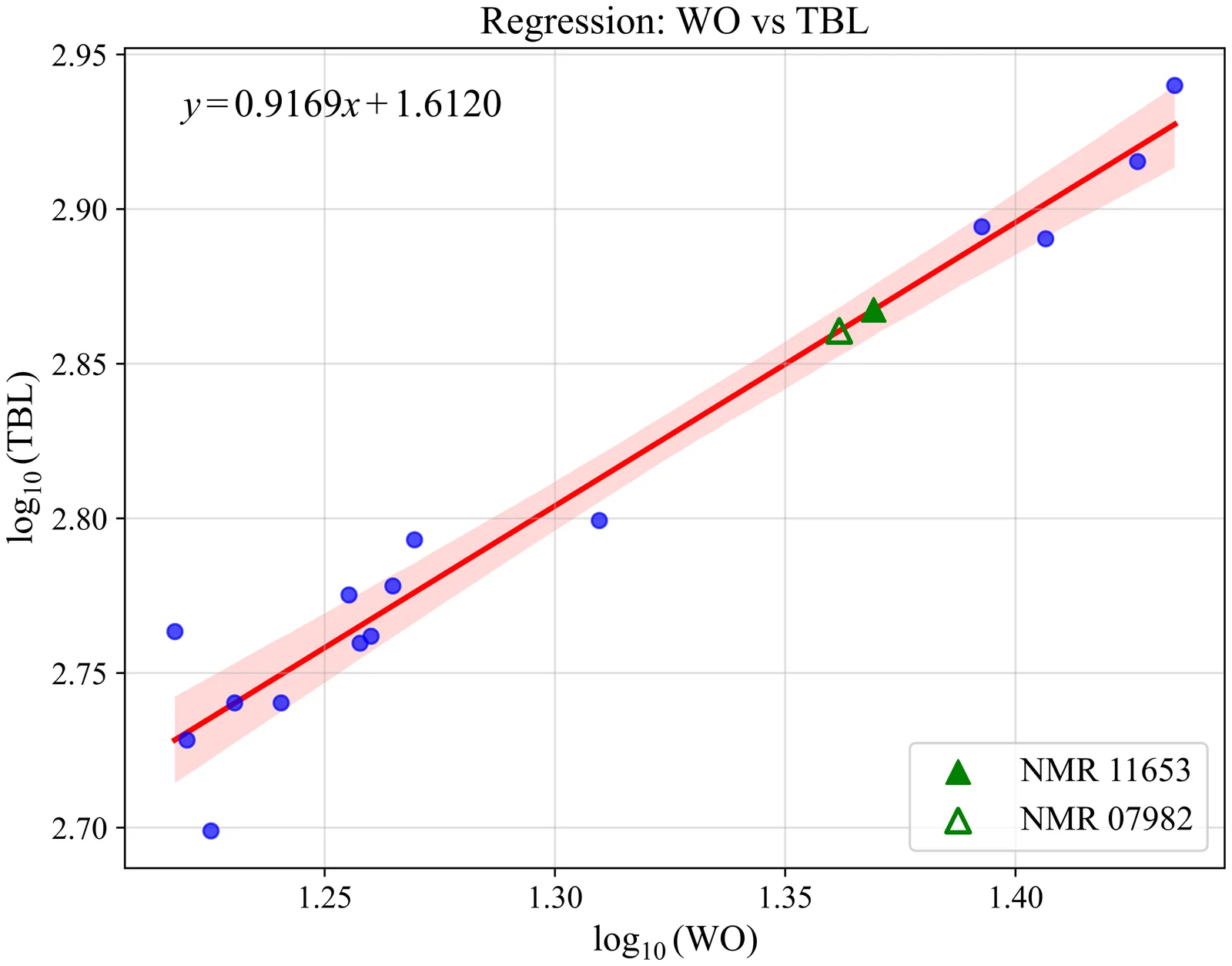
Regression analysis on atlases of *Orcinus* sp. Resulting regression of log_10_ transformed width of condylar fossae of the atlases of extant *Orcinus orca* and log_10_ transformed total body lengths for estimating the body length of NMR 07982 (hollow triangle) and NMR 11653 (filled triangle). Blue dots represent the 15 extant *Orcinus orca* included in this analysis.

Estimating body size from NMR 07979 (posterolateral left portion of cranium) and NMR 07974 (posterodorsal portion of cranium) is more challenging due to the fragmentary nature of these specimens and the substantial intraspecific variability observed in extant delphinids, partly related to ontogeny (Takahashi, Nakamura & Kato, 2021), in these cranial regions. However, rough estimates of the body size of these two killer whales were obtained using the 3D model of *O. orca* MSNUP C-301 as a comparison (Fig. 9). The original body length of MSNUP C-301 was estimated at 5.96 m, based on the skeletal length of 5.45 m (according to Braschi, Cagnolaro & Nicolosi, 2007) plus 51 cm of the estimate of the total length of the intervertebral disks based on Long et al. (1997, tab. 2).

**Figure 9:**
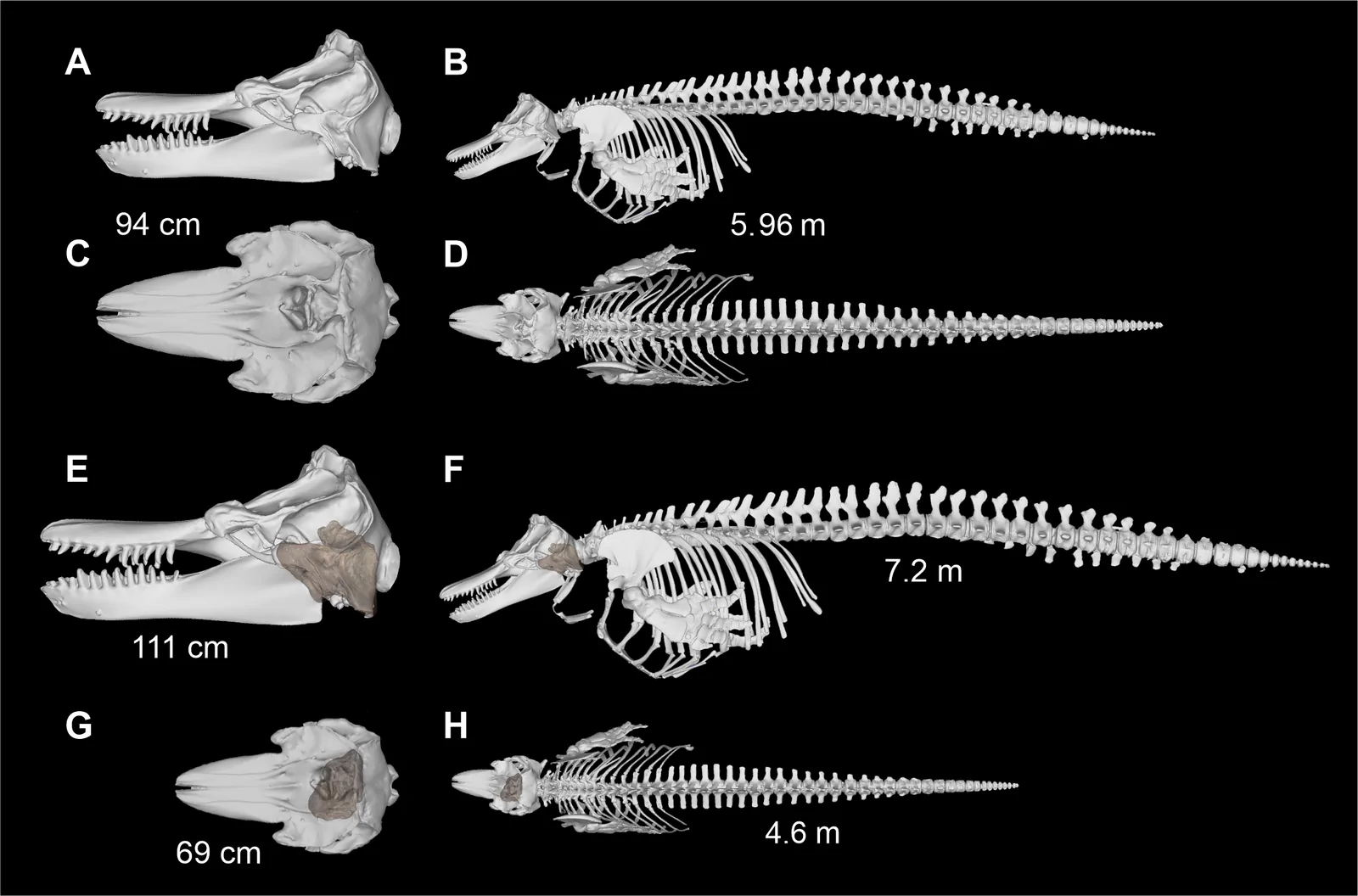
3D-based body length estimation of Gelasian *Orcinus* sp. (A–D) Reference 3D model of the skull (A, C) and complete skeleton (B, D) of the extant *Orcinus orca* (MSNUP C-301, body length 596 m). (E–F) Digital superimposition of cranial fragment NMR 07979 onto the skull (E) and complete skeleton (F) of the MSNUP C-301 model. G-H, digital superimposition of cranial fragment NMR 07974 onto the skull (G) and complete skeleton (H) of the MSNUP C-301 model skulls are shown at a common scale, and complete skeletons are shown at a common scale that is reduced relative to the skulls. A, B, E, F: left lateral views; C, D, G, H: dorsal views.

Specifically, the 3D model of both the NMR 07979 and NMR 07974 fragmentary crania were first digitally superimposed onto the corresponding anatomical regions of the MSNUP C-301 cranium. This procedure allowed us to estimate their skull lengths based on the known dimensions of MSNUP C-301. Subsequently, the complete 3D model of the MSNUP C-301 skeleton was used to extrapolate total skeletal length for the fossil specimens.

Using this approach, we obtained approximate total body length estimates of 7.2 m for NMR 07979 and 4.6 m for NMR 07974. The estimate for NMR 07979 is consistent with that obtained for NMR 07982, whereas the estimate for NMR 07974 is substantially smaller than those of the other specimens.

Regarding NMR 07979, it should be noted that the preserved cranial region exhibits positive allometry relative to the fully developed cranium (Takahashi, Nakamura & Kato, 2021), suggesting that the reconstructed skull length of 111 cm may represent an overestimate. Conversely, because skull length increases more slowly than postcranial length during ontogeny (Takahashi, Nakamura & Kato, 2021), these opposing allometric trends may partially offset each other, and the resulting body length estimate may approximate the true value.

In contrast, the skull length estimated for NMR 07974 (69 cm) is likely more reliable, as this cranial region shows isometric growth (Takahashi, Nakamura & Kato, 2021). However, given the negative allometry of skull length relative to total skeletal length, the overall body size of NMR 07974 may, in fact, have been smaller than the estimate proposed here.

Our results indicate that the Gelasian killer whale had a greater body length than the mean body length of modern orcas (near six m, see Fig. 6), although likely not surpassing extant orcas of the Type A ecotype.

## Palynological Analysis

### Facies

The association is very rich in palynomorphs (*i.e.,* microflora consisting of spores, pollen and dinoflagellate cysts). The bisaccate pollen (gymnosperms) are dominating the assemblage (49% of the total palynomorph sum and 86% of the sporomorph category). All sporomorphs comprise 57% of the total palynospectrum, hence high continental influence is recorded here. However, gymnosperms have a high buoyancy in both water and in the air. These pollen grains may be transported over large distances from their original source (upland vegetation). The marine component is dominated by the open-marine genus *Spiniferites* (49% of the total sum dinocysts). Striking is the high number of cold-tolerant marine genera and taxa (nearly 30%), like *Brigantedinium*, *Bitectatodinium tepikiense*, *Filisphaera filifera*, and *Habibacysta tectata*. Freshwater algae, *Pediastrum*, are very rare, in addition, however, a few freshwater to brackish algae, *Botryococcus*, are recorded. Reworking from older successions is rare. The association roughly indicates shallow marine conditions with proto-deltaic influence. The sea-surface temperature was temperate to cold.

### Age assessment

Although the microflora is *very* rich, not a single dinoflagellate cyst with a LOD in the Pliocene is recorded (like *e.g.*, any species of the genus *Barssidinium*, *Invertocysta lacrymosa*, and *Melitasphaeridium choanophorum*). The oldest possible age is inferred from the absence of Pliocene dinoflagellate cyst species. *Habibacysta tectata* has a LOD in the Early Pleistocene, Gelasian, at approximately 2.1 Ma. Hence, the association of the present *Orcinus* sample, taken from NMR 07979, is interpreted as Early Pleistocene, Gelasian, interval 2.6–2.1 Ma. The common number of cold tolerant marine genera and taxa fits very well with the dating.

## Discussion

Over the course of their evolutionary history, several cetacean lineages independently developed adaptations for macropredation, evolving convergent traits such as large teeth, a broad temporal fossa, and medium- to large-body size. Comparable morphologies are documented in the protocetid *Phiomicetus* from the Middle Eocene of Egypt (Gohar et al., 2021), in some basilosaurids from the Middle to Late Eocene (Uhen, 2018), and among odontocetes, in the Oligocene archaic odontocete *Ankylorhiza tiedemani* (Boessenecker et al., 2020) and several Miocene physeteroids, including *Livyatan melvillei*, *Acrophyseter* spp., and *Zygophyseter varolai*.

A link between the evolution of baleen whale body size and the Neogene to Pleistocene history of cetacean macropredation has been hypothesized. The radiation of macropredatory physeteroids occurred at a time when most baleen whales did not exceed body lengths of roughly 10 m, rendering them suitable prey for large toothed whales and sharks (Lambert et al., 2010). Sustained predation pressure from these apex predators may have favored a progressive increase in body size among baleen whales, culminating in the onset of gigantism around 3.5 Ma. This trend, also linked to the intensification of upwelling and the resulting redistribution of coastal food resources (Slater, Goldbogen & Pyenson, 2017; Bianucci et al., 2019), was accompanied not only by the evolution of large-bodied mysticetes, but also by an apparent selective decline of smaller-bodied baleen whale taxa, resulting in a restructuring of mysticete diversity. The combined effect of increasing prey size and the reduced availability of smaller mysticetes may have contributed to the decline of macropredatory physeteroids and could help explain the apparent post-Miocene drop in macropredatory odontocetes.

Among Delphinidae, macropredation has generally been considered a relatively recent phenomenon. Indeed, the putative ancestors of the two extant macropredatory species, *Orcinus orca* and *Pseudorca crassidens*, namely *Orcinus citoniensis* from the Italian Pliocene and *Rododelphis stamatiadisi* from the Pleistocene of Rhodes, have been interpreted as primarily piscivorous rather than predators of other tetrapods (Bianucci et al., 2022; Citron et al., 2022). Furthermore, until now, no significant fossil remains attributable to the two extant macropredatory delphinid species have been documented. Genomic analyses of *Orcinus orca* revealed the divergence of five major ecotypes, suggesting a relatively recent global radiation estimated at less than 250 ka and placing the origin of living ecotypes in the Middle Pleistocene (Chibanian; 0.77–0.12 Ma) (Foote et al., 2016). Consequently, the data available until now suggested a gap of approximately 5 million years, from the end of the Miocene to the Middle Pleistocene, during which no clear evidence of cetacean macropredators had been documented. It has been proposed that during the Pliocene, the ecological role of cetacean macropredators was at least partly filled by large lamniform sharks, including *Otodus megalodon*, *Cosmopolitodus plicatilis*, and *Parotodus benedeni*, which became extinct during or by the end of the Pliocene (Boessenecker et al., 2019; Citron et al., 2022; Lambert et al., 2026). Moreover, the globally decreasing sea temperatures confined extant thermophilic sharks to the tropics and reduced predation pressure at mid and high latitudes (Citron et al., 2022; Collareta et al., 2023a; Collareta, Casati & Di Cencio, 2023b).

The North Sea Gelasian killer whale described here supports the hypothesis that cetaceans reoccupied the macropredatory niche by the Early Pleistocene, well before the divergence of modern *O. orca* ecotypes. As discussed above, the large size of the alveoli and the inferred presence of long tooth roots observed in NMR 07973 suggest a macroraptorial feeding ecology. This interpretation is further supported by the relatively elongated and robust mandibular symphysis, a condition uncommon among delphinids. In addition, the deep occipital articular facets and the well-developed ridge along the ventral margin of the atlas observed in both NMR 07982 and NMR 11653 indicate an increased range of dorsoventral movement at the atlanto-occipital joint compared with most other delphinids. The ventral ridge is absent in *G. griseus* and *P. electra*, less pronounced in *G. melas* and *R. stamatiadisi*, variable in *P. crassidens* and present in *O. orca* and *F. attenuata* pointing to a greater mobility of the head which is associated with macrophagous feeding behaviour as is demonstrated by Okamura & Fujiwara (2020).

A further support to the macropredatory behaviour of the Gelasian North Sea killer whale is its large body size estimate (7.2–7.7 m) that is consistent with those of the extant killer whale populations predominantly targeting large prey including baleen whales (Pitman & Ensor, 2003; Pitman, Perryman & Eilers, 2007; Foote et al., 2009; Foote et al., 2016).

Several factors may have facilitated this ecological shift within the *Orcinus* lineage, including a decline of macropredatory sharks and, paradoxically, the extreme gigantism attained by baleen whales. The complex social structure and coordinated hunting strategies of delphinids enable cooperative attacks on large cetaceans, including the blue whale *Balaenoptera musculus* (Totterdell et al., 2022) and other mysticetes (*e.g.*, (Sironi et al., 2008; MacKay et al., 2025), thereby providing access to a substantial and energetically rewarding food resource.

## Conclusion

The five delphinid fossils described here indicate that a large killer whale was already present in the Southern North Sea Basin during the Gelasian (earliest Pleistocene). The estimated body length of this killer whale was comparable to that of the largest extant male *Orcinus orca*. The morphology of the mandible, alveoli and atlas complex support the interpretation of a macrophagous lifestyle (Fig. 10).

**Figure 10:**
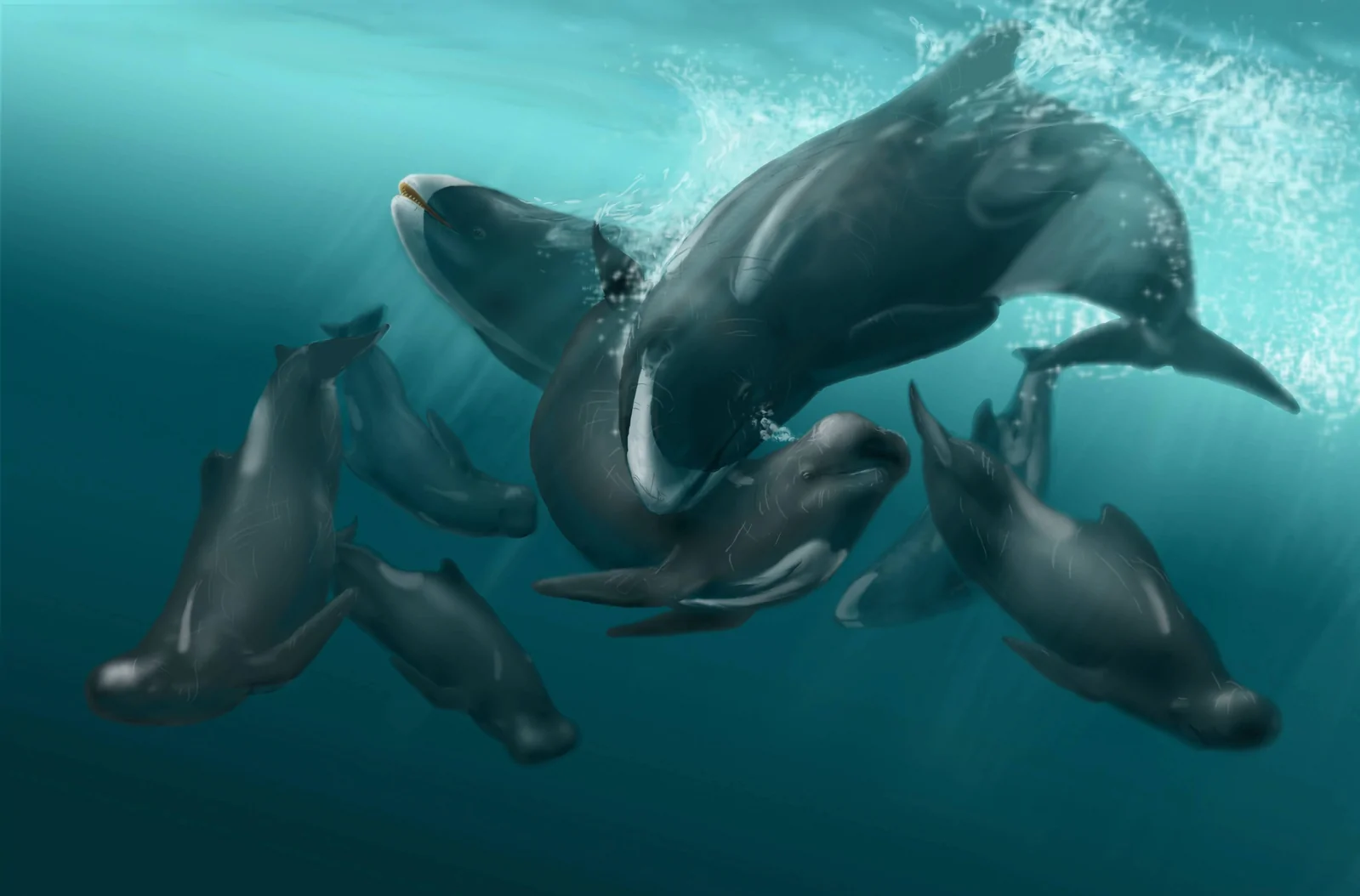
Artist reconstruction. The Early Pleistocene macropredatory *Orcinus* sp. hunting the delphinid *Platalearostrum hoekmani*. By Remie Bakker.

The fragmentary nature of the available material prevents a definitive taxonomic assignment to the species level. It is still unclear whether the Gelasian *Orcinus* represents an early presence of *O. orca* or a distinct, large, currently undescribed species within the genus. Future discoveries of more complete material will be necessary to determine its exact systematic position.

Our results indicate that large body size and macropredatory adaptations in delphinids already evolved during or before the earliest Pleistocene and this behaviour predates the emergence of modern ecotypes in the *Orcinus* lineage.

Placing the emergence of large-bodied macropredation in *Orcinus* in the earliest Pleistocene provides a deep-time perspective on the ecological role of killer whales as structuring marine food webs. Historical industrial whaling, coupled with overfishing, pollution, and ongoing climate warming, has profoundly altered ocean ecosystems, in some regions forcing *O. orca* to shift their diet from large whales to smaller prey such as pinnipeds, with cascading effects on other marine organisms (Estes et al., 2009; Roman et al., 2014). Reports of recent reductions in body size in some modern killer whale populations (Fearnbach et al., 2011) further suggest that even top predators with a multimillion-year history of macropredation are not immune to rapid anthropogenic changes. From this perspective, the Gelasian record highlights both the antiquity of macropredatory adaptations in delphinids and the remarkable evolutionary resilience of the *Orcinus* lineage, while also emphasizing that ecological strategies maintained over millions of years may nevertheless be challenged by the unprecedented pace of human-driven restructuring of marine ecosystems.

##  Supplemental Information

10.7717/peerj.21890/supp-1 Supplemental Information 1 NMR 07974 posterodorsal portion of cranium (a, b) and NMR 07979 posterolateral portion of cranium (c, d, e) *Orcinus sp.* in a, posterior view; b, dorsal view; c, ventral view; d, lateral view; e, posterolateral view.

10.7717/peerj.21890/supp-2 Supplemental Information 2 NMR 11653 (left) and NMR 07982 (right) * Orcinus sp.* fused atlas and axis vertebrae in a/d, anterior view; b/e, posterior view and c/f, dorsal view.

10.7717/peerj.21890/supp-3 Supplemental Information 3 Dinoflagellate cysts identification of a sample taken of NMR 7979 Identification of a sample taken of NMR 7979 placed in a range chart

10.7717/peerj.21890/supp-4 Supplemental Information 4 Raw data: Measurements (cm, log_10_ transformed) of the mandible of extant orcas, for regression analysis LS, greatest length of mandibular symphysis; WS, greatest width of symphysis from anteroventral to the most posterodorsal margin; aLs, anteroposterior length of symphysis; HM, dorsoventral height of mandible directly posterior of mandibular symphysis; A, distance between anterior wall of the 3th alveoli to the most posterior wall of the 9th alveoli; T3-T9, distance between the anterior wall and the posterior wall of the 3th to 9th alveoli; TBL, total body length; GM, Geometric mean.

10.7717/peerj.21890/supp-5 Supplemental Information 5 Predictive regression equations for the mandible LS, greatest length of mandibular symphysis; WS, greatest width of symphysis from anteroventral to the most posterodorsal margin; aLs, anteroposterior length of symphysis; HM, dorsoventral height of mandible directly posterior of mandibular symphysis; A, distance between anterior wall of the 3e alveoli to the most posterior wall of the 9e alveoli; T3-T9, distance between the anterior wall and the posterior wall of the 3 th to 9 th alveoli; TBL, total body length; GM, Geometric mean.

10.7717/peerj.21890/supp-6 Supplemental Information 6 Raw data: Measurements (cm, log_10_ transformed) of atlases of extant orcas, for regression analysis WO, greatest width of condylar fossae; TBL, total body length.

10.7717/peerj.21890/supp-7 Supplemental Information 7 Predictive regression equations for the atlas vertebrae WO, greatest width of condylar fossae; TBL, total body length.

10.7717/peerj.21890/supp-8 Supplemental Information 8 Raw data regression analysis table 1 LS, greatest length of mandibular symphysis; WS, greatest oblique height of symphysis from anteroventral to the most posterodorsal margin; aLs, anteroposterior length of symphysis; HM, dorsoventral height of mandible directly posterior of mandibular symphysis; A, distance between anterior wall of the 3th alveoli to the most posterior wall of the 9th alveoli; T3–T9 anteroposterior measurements of alveoli; TBL, total body length measured during stranding of specimen.

10.7717/peerj.21890/supp-9 Supplemental Information 9 Raw data regression analysis table 2 WO, maximum of occipital articular facets; NA, distance between ventral border of neural canal and ventral border of atlas vertebra; WN, width of neural canal measured at the base of the pedicles; TBL, total body length

## Institutional abbreviations

STME: Stichting Texels Museum Ecomare, De Koog, Texel, The Netherlands.
IRSNB: Institut royal des Sciences naturelles de Belgique, Brussels, Belgium.
MGGC: Collezione di Geologia “Museo Giovanni Capellini”, Università di Bologna, Italy.
MMPS: Stamatiadis Museum of Mineralogy and Paleontology, Rhodes, Greece.
MSNUP: Museo di Storia Naturale, Università di Pisa, Italy.
NBC: Naturalis Biodiversity Center, Leiden, The Netherlands.
NHMUK: Natural History Museum, London, United Kingdom.
NMR: Natuurhistorisch Museum Rotterdam, The Netherlands.
NMNZ: Museum of New Zealand Te Papa Tongarewa, Wellington, New Zealand.
RMNH: Rijksmuseum van Natuurlijke Historie, Leiden, The Netherlands (now part of NBC).
SMNK: State Museum of Natural History, Karlsruhe, Germany.
TNO-GSN: TNO-Geological Survey of the Netherlands.
UABCS: Museo de Historia Natural, Universidad Autónoma de Baja California Sur, La Paz, Baja California Sur, Mexico.
USNM: National Museum of Natural History, Smithsonian Institution, Washington, USA.
ZMA: Zoölogisch Museum Amsterdam, Amsterdam, The Netherlands (now part of NBC).

## Additional Information and Declarations

### Data Availability

The following information was supplied regarding data availability: The data is available in the Supplemental Files. The Orcinus orca skeleton is available in the collection of Natural History Museum University of Pisa under number: MSNUP C-301:
https://sketchfab.com/3d-models/killer-whale-5be39567f8624064be904662b6813fd2
3D scans are available on MorphoSource: - Dorsal cranium fragment: NMR 07974: Showcase Media // MorphoSource
https://doi.org/10.17602/M2/M842064
- Posterolateral portion of cranium: NMR 07979: Showcase Media // MorphoSource
https://doi.org/10.17602/M2/M842056.
