# Supplementary material for: A large orca from the Lower Pleistocene of the Southern North Sea Basin pushes back the origin of macropredatory behavior in the oceanic dolphins (Odontoceti, Delphinidae)
Source: PeerJ. 2026 Nov 10;14:e21890. doi: 10.7717/peerj.21890 (PMC13762349; doi:10.7717/peerj.21890)
Supplement: Supplementary file 1 [file peerj-14-21890-s004.docx]

| Spec | LS(A) | WS(B) | aLs(C) | HM(D) | A(E) | T3 | T4 | T5 | T6 | T7 | T8 | T9 | TBL | GM |
| --- | --- | --- | --- | --- | --- | --- | --- | --- | --- | --- | --- | --- | --- | --- |
| ZMA.MAM.  13492 | 1.18 | 0.81 | 1.18 | 0.94 | 1.25 | 0.20 | 0.23 | 0.32 | 0.20 | 0.23 | 0.20 | 0.23 | 2.70 | 0.58 |
| RMNH.MAM. 17711 | 1.15 | 0.83 | 1.13 | 0.93 | 1.27 | 0.18 | 0.28 | 0.26 | 0.30 | 0.28 | 0.23 | 0.26 | 2.70 | 0.59 |
| NHMUK ZC. 1934.7.23.2 | 1.31 | 0.94 | 1.26 | 1.18 | 1.44 | 0.45 | 0.49 | 0.52 | 0.52 | 0.52 | 0.49 | 0.49 | 2.88 | 0.80 |
| NHMUK ZC. 1917.10.2.1 | 1.31 | 0.98 | 1.30 | 1.14 | 1.40 | 0.52 | 0.52 | 0.53 | 0.53 | 0.51 | 0.48 | 0.45 | 2.89 | 0.81 |
| NHMUK SW. 1918/10 | 1.20 | 0.83 | 1.20 | 1.02 | 1.31 | 0.34 | 0.36 | 0.32 | 0.32 | 0.36 | 0.32 | 0.28 | 2.79 | 0.66 |
| NHMUK SW.  1918.12 | 1.21 | 0.82 | 1.16 | 0.97 | 1.28 | 0.32 | 0.40 | 0.41 | 0.34 | 0.32 | 0.28 | 0.26 | 2.79 | 0.65 |
| NHMUK SW. 2001/234 | 1.21 | 0.80 | 1.19 | 0.96 | 1.29 | 0.34 | 0.36 | 0.38 | 0.38 | 0.36 | 0.34 | 0.32 | 2.77 | 0.66 |
| NHMUK SW.  1943/9 | 1.22 | 0.83 | 1.21 | 0.92 | 1.33 | 0.38 | 0.40 | 0.41 | 0.38 | 0.36 | 0.32 | 0.32 | 2.81 | 0.67 |
| NHMUK SW.  1943-11 | 1.21 | 0.88 | 1.20 | 0.94 | 1.33 | 0.43 | 0.36 | 0.40 | 0.41 | 0.38 | 0.38 | 0.36 | 2.80 | 0.69 |
| NHMUK SW. 1937/27 |  |  |  | 0.98 | 1.32 | 0.40 | 0.36 | 0.36 | 0.36 | 0.34 | 0.30 | 0.28 | 2.82 |  |
| NHMUK SW. 1944/2 | 1.26 | 1.02 | 1.25 | 1.06 | 1.40 | 0.43 | 0.41 | 0.45 | 0.48 | 0.49 | 0.48 | 0.45 | 2.90 | 0.77 |
| NMR  9990000933 | 1.12 | 0.82 | 0.96 | 0.97 | 1.25 | 0.20 | 0.28 | 0.30 | 0.32 | 0.28 | 0.23 | 0.23 | 2.70 | 0.58 |
| RMNH.MAM.  7514 | 1.16 | 0.87 | 1.15 | 0.92 | 1.19 | 0.26 | 0.26 | 0.28 | 0.30 | 0.32 | 0.30 | 0.26 | 2.76 | 0.60 |
| RMNH.MAM.  1487 | 1.18 | 0.83 | 1.16 | 0.98 | 1.19 | 0.34 | 0.30 | 0.30 | 0.32 | 0.34 | 0.23 | 0.23 | 2.79 | 0.62 |
| RMNH.MAM.  13487 | 1.16 | 0.76 | 1.10 | 0.95 | 1.18 | 0.28 | 0.34 | 0.34 | 0.32 | 0.30 | 0.30 | 0.28 | 2.73 | 0.61 |
| RMNH.MAM.  31219 | 1.20 | 0.88 | 1.17 | 0.94 | 1.25 | 0.34 | 0.36 | 0.36 | 0.36 | 0.34 | 0.34 | 0.30 | 2.76 | 0.65 |
| ZMA.MAM.  4211 | 1.15 | 0.80 | 1.07 | 0.83 | 1.30 | 0.32 | 0.34 | 0.34 | 0.36 | 0.36 | 0.34 | 0.30 | 2.74 | 0.63 |
| RMNH.MAM.  1982 | 1.16 | 0.87 | 1.13 | 0.96 | 1.21 | 0.41 | 0.28 | 0.30 | 0.28 | 0.32 | 0.30 | 0.26 | 2.76 | 0.62 |
| Ecomare B2-09 | 1.19 | 0.85 | 1.20 | 0.92 | 1.29 | 0.32 | 0.30 | 0.30 | 0.34 | 0.30 | 0.28 | 0.28 | 2.74 | 0.63 |
