# Supplementary material for: A large orca from the Lower Pleistocene of the Southern North Sea Basin pushes back the origin of macropredatory behavior in the oceanic dolphins (Odontoceti, Delphinidae)
Source: PeerJ. 2026 Nov 10;14:e21890. doi: 10.7717/peerj.21890 (PMC13762349; doi:10.7717/peerj.21890)
Supplement: Supplementary file 2 [file peerj-14-21890-s005.docx]

| Equation | Regression Equation | Adjusted R² | p-value | %PE | %SEE |
| --- | --- | --- | --- | --- | --- |
| TBL~LS(A) | y = 1.048x + 1.52 | 0.79 | <0.001 | 4.87 | 1.00 |
| TBL~WS(B) | y = 0.752x + 2.134 | 0.65 | <0.001 | 6.95 | 1.30 |
| TBL~aLS(C) | y = 0.621x + 2.053 | 0.58 | <0.001 | 6.49 | 1.42 |
| TBL~HM(D) | y = 0.556x + 2.238 | 0.52 | <0.001 | 7.15 | 1.49 |
| TBL~A(E) | y = 0.619x + 1.983 | 0.54 | <0.001 | 6.99 | 1.46 |
| TBL~T3 | y = 0.584x + 2.581 | 0.76 | <0.001 | 4.91 | 1.07 |
| TBL~T4 | y = 0.673x + 2.545 | 0.69 | <0.001 | 5.78 | 1.20 |
| TBL~T5 | y = 0.652x + 2.543 | 0.67 | <0.001 | 6.24 | 1.24 |
| TBL~T6 | y = 0.636x + 2.551 | 0.71 | <0.001 | 6.27 | 1.16 |
| TBL~T7 | y = 0.727x + 2.522 | 0.85 | <0.001 | 3.92 | 0.84 |
| TBL~T8 | y = 0.611x + 2.582 | 0.71 | <0.001 | 5.86 | 1.17 |
| TBL~T9 | y = 0.642x + 2.583 | 0.67 | <0.001 | 6.82 | 1.24 |
| TBL~Geo_Mean | y = 0.829x + 2.233 | 0.88 | <0.001 | 4.12 | 0.77 |
