# Supplementary material for: A large orca from the Lower Pleistocene of the Southern North Sea Basin pushes back the origin of macropredatory behavior in the oceanic dolphins (Odontoceti, Delphinidae)
Source: PeerJ. 2026 Nov 10;14:e21890. doi: 10.7717/peerj.21890 (PMC13762349; doi:10.7717/peerj.21890)
Supplement: Supplementary file 3 [file peerj-14-21890-s006.docx]

| Spec | WO | TBL |
| --- | --- | --- |
| NHMUK ZC. 1934.7.23.2 | 1.43 | 2.94 |
| NHMUK ZC. 1924.11.4.1 | 1.39 | 2.89 |
| NHMUK ZC. 1924.11.4.2 | 1.43 | 2.92 |
| NHMUK ZC. 1917.10.2.1 | 1.41 | 2.89 |
| RMNH.MAM.17711 | 1.23 | 2.70 |
| RMNH.MAM.7514 | 1.26 | 2.76 |
| RMNH.MAM.1487 | 1.27 | 2.79 |
| RMNH.MAM.13487 | 1.22 | 2.73 |
| RMNH.MAM.31219 | 1.22 | 2.76 |
| RMNH.MAM.1982 | 1.26 | 2.76 |
| ZMA.MAM.4211 | 1.24 | 2.74 |
| NMR9990000933 | 1.26 | 2.78 |
| Ecomare B2-09 | 1.23 | 2.74 |
| MSNUP C-298 | 1.31 | 2.80 |
| MSNUP C-301 | 1.26 | 2.78 |
