# Supplementary material for: A large orca from the Lower Pleistocene of the Southern North Sea Basin pushes back the origin of macropredatory behavior in the oceanic dolphins (Odontoceti, Delphinidae)
Source: PeerJ. 2026 Nov 10;14:e21890. doi: 10.7717/peerj.21890 (PMC13762349; doi:10.7717/peerj.21890)
Supplement: Supplementary file 4 [file peerj-14-21890-s007.docx]

| Spec | X_Variable | Regression_Equation | Adjusted_R² | P-value | %SEE | %PE |
| --- | --- | --- | --- | --- | --- | --- |
| NMR_11653 | TBL~WO | y = 0.917x + 1.612 | 0.95 | <0.001 | 0.60 | 2.72 |
| NMR_07982 | TBL~WO | y = 0.917x + 1.612 | 0.95 | <0.001 | 0.60 | 2.72 |
